# A database to initiate methodological advances in the evaluation of transitivity assumption in network meta-analysis: qualitative features and limitations of the tracenma R package

**DOI:** 10.1186/s12874-025-02634-x

**Published:** 2025-07-31

**Authors:** Loukia M. Spineli, Andrés Mauricio García-Sierra, Juan Jose Yepes-Nuñez

**Affiliations:** 1 Midwifery Research and Education Unit (OE 9210), Hannover Medical School, Carl-Neuberg-Straße 1, Hannover, 30625 Germany; 2 Department of Public Health Sciences, University of Chicago, Chicago, USA; 3 School of Medicine, Universidad de los Andes, Bogotá, D.C Colombia; 4 Internal Medicine Department, Fundación Santa Fe de Bogotá, Bogotá, D.C Colombia

**Keywords:** Network meta-analysis, Systematic review, Transitivity assumption, R package, Database

## Abstract

**Background:**

Transitivity assumption underlies the network meta-analysis framework and states that treatment comparisons are similar regarding the distribution of important characteristics that act as effect modifiers. Currently, there is a lack of methods to assess transitivity quantitatively. The methodological gap in assessing the transitivity assumption motivated the development of the tracenma database, aspiring to initiate methodological advances in transitivity assessment using empirical data.

**Methods:**

We used the nmadb R package to build a database of connected networks and consulted the relevant literature to determine the necessary characteristics comprising potential effect modifiers. We referred to the systematic review report and supplementary material for each eligible network to retrieve at least four extractable characteristics. The extracted information comprised the studies, corresponding treatment comparisons, and aggregated characteristics at the study level. We classified the characteristics into three types (clinical, demographic and methodological) and ten subtypes (participants, treatments, outcomes, age, sex, ethnicity, study design, study setting, risk of bias and withdrawals). We summarised the distribution of the characteristics and missing data across all datasets by type and subtype.

**Results:**

Of the 453 networks in the nmadb database, 217 (48%) were eligible for the tracenma database, with each network comprising a dataset. The extracted characteristics ranged from 4 to 35 (median: 11), with the middle half comprising 8 to 15 characteristics. In most datasets, some characteristics were transformed for being reported inconsistently across the studies concerning the measure scale and summary statistics. Methodological characteristics dominated all datasets and were less prone to missing data. The study design was ubiquitous among the methodological subtypes, while withdrawals were hardly reported. Clinical characteristics were present in most datasets and were subject to most missing data; their characteristic subtypes received uneven attention, with participant characteristics reported more frequently. Demographic characteristics were the least frequently reported. Seventy per cent of the datasets included all types; however, the characteristic subtypes varied substantially in frequency.

**Conclusions:**

Tracenma is the first database built to motivate the development and empirical evaluation of novel methods for assessing transitivity. The database is hosted in the tracenma R package with functions that facilitate data access.

**Supplementary Information:**

The online version contains supplementary material available at 10.1186/s12874-025-02634-x.

## Background

Software development and data availability play a crucial role in the advancement, dissemination and reproducibility of methodology in any research area. In the evidence synthesis field, there has been a rapid increase in software of diverse operationality that facilitates the conduct, analysis and reporting of systematic reviews by fostering collaborations worldwide and assisting the authors in relevant labour-intensive tasks, including study search, identification and eligibility, data extraction, and risk of bias assessment [1, 2]. The explosion in publications of primary and secondary research of disparate quality necessitated even more the synergy of software and human effort in evidence synthesis, also leading to the development of a new generation of systematic reviews called living systematic reviews [3–5].

Radical advances in evidence synthesis methodology and broad application from users of diverse statistical knowledge are attributed, among others, to the rise in software availability for statistical analysis [6–8]. The R software has been at the forefront of evolving and disseminating state-of-the-art statistical methods and data visualisations for evidence synthesis [9, 10]. Being open-source and free software, R has cultivated an avid evidence synthesis community across disciplines, contributing to several fit-for-purpose R packages and recently hosting the online conference for evidence synthesis in R [9, 11]. Recent additions to the R library constitute R packages offering a collection of datasets for pairwise and network meta-analysis from different disciplines and targeting multiple purposes, including teaching, illustration and empirical validation of meta-analytic methods [9].

Network meta-analysis (NMA), an extension of pairwise meta-analysis that answers more complex research questions, has received great research attention, leading to a fast-paced, enriched R toolkit with packages to apply the new methodological advances on this research topic [9, 12]. Currently, nmadb is the only R package that provides a comprehensive database with datasets on various outcome types, effect measures and formats for empirical investigation of NMA models and related methods [13]. The database has been built upon the bibliographic study of Petropoulou et al. [14], who collected systematic reviews with multiple treatments published between 1999 and 2015. The nmadb database [13] has already been used in several empirical studies, such as concerning established and newly proposed ranking measures [15–17], the commonness of statistical inconsistency between different evidence sources [18], the contribution of indirect evidence in the NMA results [19], and reporting quality of transitivity assumption [20].

We recently added the tracenma R package [21] to the R toolkit for NMA, which provides a *database* with study-level aggregate clinical and methodological characteristics reported in published systematic reviews with NMA to assist in developing and empirically evaluating statistical methods for the assessment of the transitivity assumption. Transitivity posits that treatment comparisons, forming a connected treatment network, are similar regarding the distribution of important clinical and methodological characteristics that act as effect modifiers [22]. This research topic has attracted great interest in the relevant literature since the early promotion of NMA as the cornerstone of the NMA framework [22–25]. However, the published literature has revolved around the conceptual features and importance of transitivity, with stagnant progress in methods to evaluate this assumption, as opposed to other core NMA elements, such as statistical heterogeneity and inconsistency, that have been subjected to constant methodological advances [12, 26].

Transitivity is one of the dimensions used to judge the confidence level in the NMA results through the indirectness domain [27, 28]. For a ‘typical’ network with sparsely informed treatment comparisons, reliance on meta-regression to improve the plausibility of transitivity or comparing the distribution of the effect modifiers (the state of affairs in evaluating transitivity) may be problematic [27], underlying the need to develop rigorous methods for transitivity assessment. A recent methodological study proposed a novel approach to assess transitivity based on study dissimilarities for characteristics that act as important effect modifiers, tackling the aforementioned limitations [29]. This methodological study used the tracenma R package [21] to evaluate the proposed approach *empirically* [29].

The shortage of methods targeting transitivity evaluation inspired the creation of the tracenma database [21], intending to initiate the development and empirical evaluation of statistical methods to assess the transitivity assumption. The present article aims to introduce the tracenma database [21] by describing its conception and challenges during the extraction of the included datasets, summarising the qualitative features, and discussing limitations in the content of the database.

## Methods

### Building the tracenma database

The tracenma database [21] was built upon the nmadb database (version 1.2.0) [13] using sequential steps. Initially, the nmadb database [13] was restricted to treatment networks with available data on the investigated primary outcome, based on a homonym column with values ‘True’ and ‘False’ indicating whether study-level outcome data were extracted for each network, such as the number of events and randomised participants for each arm or mean difference and standard error. Hence, of the 453 networks found in nmadb [13], 286 (63%) had available data for all included studies and were considered eligible for the next step.

#### Excluded networks

Subsequently, we sought the published report and supplementary material for each eligible network. We considered a minimum threshold of four extractable characteristics per network, leading to the exclusion of 33 (12%) networks from the set of 286 eligible networks. The selected threshold of four characteristics was not based on statistical or methodological reasoning, as it was chosen arbitrarily to ensure sufficient characteristics to explore transitivity, imitating somewhat the threshold of four treatments adopted by the nmadb database [13] to include a connected network. We regard networks with fewer than four characteristics to offer insufficient information to allow for transitivity evaluation. Extractable characteristics shared the following profile: they were missing in few or none of the included studies, they were not textual, or when textual, they could be coded into distinct categories without diluting their intended clinical or methodological relevance. Twenty-five networks were further excluded for not providing any information on the included studies’ clinical and methodological characteristics. Five reports summarised the clinical and methodological characteristics per treatment or across studies (e.g., mean age in the whole network) rather than per study or treatment comparison, which would have allowed transitivity assessment; thus, the corresponding networks were excluded.

Further exclusions included one network in Hungarian, one in Chinese and one with characteristics reported in textual format. One network missing all extractable characteristics in some studies lost connectivity after excluding these studies and was not considered in the database. The nmadb database [13] did not provide any identifier for the studies of one network, such as the study names or references, making it difficult to locate these studies in the corresponding published report or supplementary material and proceed with the extraction; thus, this network was excluded. Lastly, another network was excluded due to unresponsiveness from the corresponding author to share data. In total, 217 networks comprised the tracenma database [21]. Table 1 presents the selection process of the networks to build the tracenma database [21]. Additional file 1 lists the excluded networks with reasons for exclusion.

**Table 1:** Network selection process to build the tracenma database [21]

Selection process	Networks (%)
Total networks found in the nmadb database [13]	453
Total networks excluded	236
Networks with unavailable outcome data	167 (70.8%)
Extractable characteristics were less than four	33 (14.0%)
No information on the studies’ clinical and methodological characteristics	25 (10.6%)
Characteristics were summarised per treatment or across studies	5 (2.1%)
Article in Hungarian	1 (0.4%)
Article in Chinese	1 (0.4%)
Characteristics were reported in textual format	1 (0.4%)
Network connectivity was lost after removing studies with missing data	1 (0.4%)
No study names or references were provided by nmadb [13]	1 (0.4%)
No response from the corresponding author to share the requested data	1 (0.4%)
Total networks eligible for the tracenma database [21]	217

#### Format of the datasets

Each eligible network from the set of 217 comprises a dataset with study-level aggregate clinical and methodological characteristics. Most networks found in the nmadb database [13] were in long format, where each study occupied as many rows as the number of compared treatments, with the corresponding summary statistics (e.g., number of events and randomised participants) populating one column each. We used the pairwise function of the netmeta R package [30] to turn these networks into a wide format, where each study occupied as many rows as the number of possible comparisons among the investigated treatments. For instance, a two-arm study appeared once, while a three-arm study appeared thrice in the dataset. This format conversion was necessary as transitivity assessment pertains to the similarity of treatment comparisons regarding several clinical and methodological characteristics. We maintained the information on the study identifier and compared treatments as provided in nmadb [13] but excluded the information on the outcome data for being irrelevant to the transitivity evaluation.

#### Extracted clinical, demographic and methodological characteristics

The extracted characteristics comprise potential effect modifiers commonly encountered in the literature about the set-up, conduct and analysis of systematic reviews with multiple treatments [31–34]. We distinguished the extracted characteristics into clinical, demographic and methodological. Clinical characteristics pertained to participant, treatment and outcome features. Baseline characteristics referring to comorbidities, diagnostic criteria, severity of the investigated condition, inclusion and exclusion criteria, performance status, and other relevant risk factors comprised participant-related characteristics. Information on the treatment dose, type, duration, frequency, administration, and allowed co-treatments constituted treatment-related characteristics. Scale scores and time points considered for measuring the outcome and follow-up length pertained to the outcome characteristics. We regarded age, sex and ethnicity as demographic characteristics.

Methodological characteristics included study design, study setting, risk of bias assessment and withdrawals. The study design involved sample size; planned statistical analysis (e.g., intention-to-treat or per protocol); randomisation, allocation concealment and blinding type; double-blinding period length; baseline, titration and maintenance durations; study duration; design (e.g., parallel, split-mouth, or crossover); study phase; wash-out period; enrichment enrollment and randomisation unit (e.g., participants or clusters). The study setting comprised geographic locations, being a multicenter study, receiving sponsorship or funding, and having conflicts of interest. Location was commonly reported as the country where the study was conducted; instead, we considered the corresponding continent to avoid creating too many levels for the location. Risk of bias assessment pertained to the quality of several risk of bias domains regarding the internal validity of each study based on various risk of bias tools. Lastly, withdrawals referred to the percentage of post-randomisation dropouts, lost to follow-up, and withdrawals.

### Challenges during the extraction

#### Clinical and demographic characteristics reported per treatment arm

Several systematic reviews reported numerically summarised characteristics per treatment arm rather than at the study level. Demographic characteristics, such as the percentage of females and mean age, and numeric participant characteristics, such as mean baseline HbA1c, were prone to such a reporting format. In a standard synthesis of aggregate data, effect modification pertains to the interaction of relative treatment effects with the covariate summarised at the study level concerning participant-related characteristics. Therefore, we converted these characteristics into a study-level sensible value for each reported descriptive statistic using weighted (arithmetic) mean, with weights equal to the size of each arm in the corresponding studies. For instance, if females comprised 80% and 70% of the randomised sample in the experimental and control arm of a study, the weighted mean of percentage females *for that study* would be

$$\:\frac{\left(0.80\times\:225\right)+\left(0.70\times\:227\right)}{225+227}\times\:100=75\%$$.

with 225 and 227 being the number of randomised participants in the experimental and control arm, respectively. Likewise, we would proceed, for instance, with mean age and mean baseline HbA1c when reported per arm. If the standard deviation of age is also reported per arm, we calculate the square root of the weighted mean of the variances. For example, in the study above, if the standard deviation of age were 0.5 and 0.7 for the experimental and control arm, the ‘summarised’ standard deviation *for that study* would be

$$\:\sqrt{\frac{\left({0.5}^{2}\times\:225\right)+\left({0.7}^{2}\times\:227\right)}{225+227}}=0.61$$.

Only a handful of datasets summarised age or participant characteristics per sex, such as the average age was 30 years for females and 40 for males. In this case, the conversion above was employed to summarise the age at the study level, with weights being the number of females and males in the corresponding studies.

Though mostly reported for each arm, treatment characteristics did not undergo the conversions above because these characteristics are tailored to the investigated treatments. Therefore, the dose, duration and administration will differ from treatment to treatment and may also differ across the studies investigating the same treatment comparison. An exception was co-treatments allowed, or previous treatments received when reported as the percentage of participants for each arm; in this case, the conversions above were employed.

Outcome characteristics were mainly reported at the study level and included qualitative characteristics. However, the conversions above were implemented when outcome characteristics were reported per arm, such as mean scale score at baseline. Overall, 115 datasets out of 217 (53%) underwent the conversions above for at least one demographic, participant or outcome characteristic summarised in percentage or other descriptive statistics. By definition, methodological characteristics are tailored to the study and reported at the study level; hence, no conversion was required.

#### Characteristics reported with inconsistent measures or statistics and other limitations

A common theme in many extracted datasets was summarising a numeric characteristic (typically age and participant characteristics) inconsistently across the studies using different descriptive statistics. We opted for consistent reporting across the studies by converting to the descriptive statistic used in most studies for that characteristic. For example, if few studies summarised baseline HbA1c with range bounds and most studies reported the standard deviation of baseline HbA1c, we applied formula (9) in Wan and colleagues [35] to convert the range bounds into standard deviation. Note that when these range bounds were reported per arm, we converted them into standard deviation for each arm, then proceeded with the weighted mean to obtain the ‘summarised’ standard deviation for the corresponding studies. Table 2 summarises the descriptive statistics and implemented conversions for the scenarios encountered.

**Table 2:** Conversions of descriptive statistics for consistency across studies

Mostly reported	Fewer studies reported	Converted into (and assumption made)
mean	range bounds^a^	median and assumed to coincide with the mean
	median with range bounds^a^	mean using formula (3) in [35]
	median	assumed to coincide with the mean
median	mean	assumed to coincide with the median
standard deviation	range bounds^a^	standard deviation using formula (9) in [35]
	interquartile range	standard deviation using formula (15) in [35]
range bounds^a^	mean with standard deviation	solving the system of formulas (3) and (9) in [35]
	mean	replaced with *NA* to be considered missing

^a^Range bounds refer to the minimum and maximum

Duration and follow-up length were often reported inconsistently across studies, such as mixing days with weeks, months, or years. In line with the above, we transformed into the measure reported in most studies. Furthermore, follow-up length was reported per treatment arm for some studies, and we considered the mean across the arms.

There was also inconsistency in the reported sample size in some datasets, with many studies reporting the randomised and others the assessed sample size. When, for some studies, the total sample size was reported, and the conversions mentioned above were required, we used the sample size per arm as found in nmadb [13]. We also referred to nmadb [13] when the sample size was not reported. Hence, the extracted sample size may not reflect a randomised sample or be a mix of randomised and assessed participants in some datasets.

Dose was also subject to inconsistent reporting across the studies; however, conversions to common reporting did not apply to all cases. Conversion to a common measure was possible where, for instance, the milligram dose was mixed with the percentage of grams across the studies, such as in Mason and colleagues [36]. Contrariwise, conversion into milligrams was not feasible for the studies that reported the dose in milligrams per kilogram. Overall, treatment characteristics received the least conversions since dose-related features inherently differ across the investigated treatments. In a few datasets, where the dose was reported as a range in some studies, we chose the bound considered in most studies investigating the same treatments.

For some studies, reporting numeric characteristics using only one or both range bounds was relatively common in many datasets. When one bound was reported, such as adults over 20 or up to 60 years old, we considered the reported bound, probably instigating spurious variability for these characteristics. When both bounds were reported, we considered the bound used in most studies investigating the corresponding treatment comparison. Likewise, for a few studies that reported two or more levels of a characteristic, such as diagnostic criteria, we considered only the level reported from most studies investigating the same treatment comparison.

In most datasets with multi-arm studies, participant and demographic characteristics were reported for the whole study rather than per treatment comparison. Since transitivity evaluation pertains to treatment comparisons, we considered these values for all comparisons in these studies, a defensible assumption for randomised controlled studies.

Lastly, we encountered typos in a few characteristics for some studies. However, most of the corresponding reports did not provide the studies in the reference list or the supplementary material to allow for the correction of the typos, increasing the risk of spurious variability for these characteristics.

### Data analysis on the qualitative features of the datasets

#### Describing the distribution and commonness of the main dataset features

We used median, interquartile range (IQR) and range to summarise the network features, including the number of studies, treatments and observed comparisons, and the number of characteristics for each *type* (clinical, demographic and methodological) and *subtype* (participant, treatment, outcome, age, sex, ethnicity, study design, study setting, risk of bias, and withdrawals). We illustrated the distribution of the number of characteristics for each type and subtype using box plots with integrated dots. We recorded the number of characteristics found in each dataset and used a line plot to present their frequency for each characteristic type across the datasets. The number of datasets containing at least one characteristic from the different types and subtypes was summarised using grouped bar plots. We counted the frequency of observing (i) all three characteristic types, (ii) pairs of two and (iii) only one characteristic type across the datasets and presented the results using grouped bar plots. A dataset including all characteristic types was regarded as *potentially complete*; hence, we investigated the commonness of these datasets in the tracenma database [21].

#### Describing the distribution and commonness of missing data

We summarised the distribution of missing data in two dimensions: (i) the percentage of characteristics with at least one missing data and (ii) the percentage of missing data across the datasets. We used box plots with integrated dots to illustrate the percentage of characteristics with at least one missing data per characteristic type and subtype.

#### Statistical software

All analyses were performed in the statistical software R (version 4.3.2 [37]). We used the ggplot2 R package [38] to create the figures and the tracenma R package [21] to access the database with 217 datasets on the study-level summarised characteristics. A description of the functionalities of the tracenma R package [21] is available at https://loukiaspin.github.io/tracenma/ [39]. Additional file 2 presents a tutorial on the synergy of the tracenma R package [21] and currently available methods to assess transitivity.

## Results

### Describing the distribution and commonness of the main dataset features

#### Network-related features

The datasets varied notably in the number of studies and treatments, including 5 to 145 studies (median: 17) and 3 to 40 treatments (median: 7); however, the middle half of the datasets covered a substantially smaller number of studies and treatments (IQR: 11–29 and IQR: 5–9, respectively). In the nmadb database [13], the selected primary outcome was not extracted for three studies in one network with four treatments. After excluding these studies, one treatment was dropped from the network without affecting network connectivity; hence, the minimum value of 3 treatments. The percentage of observed treatment comparisons ranged from 7 to 100% (median: 42%, IQR: 32–53%), indicating diversity in network geometry, with three networks having all possible treatment comparisons observed.

#### Characteristic-related features

**Fig. 1:**
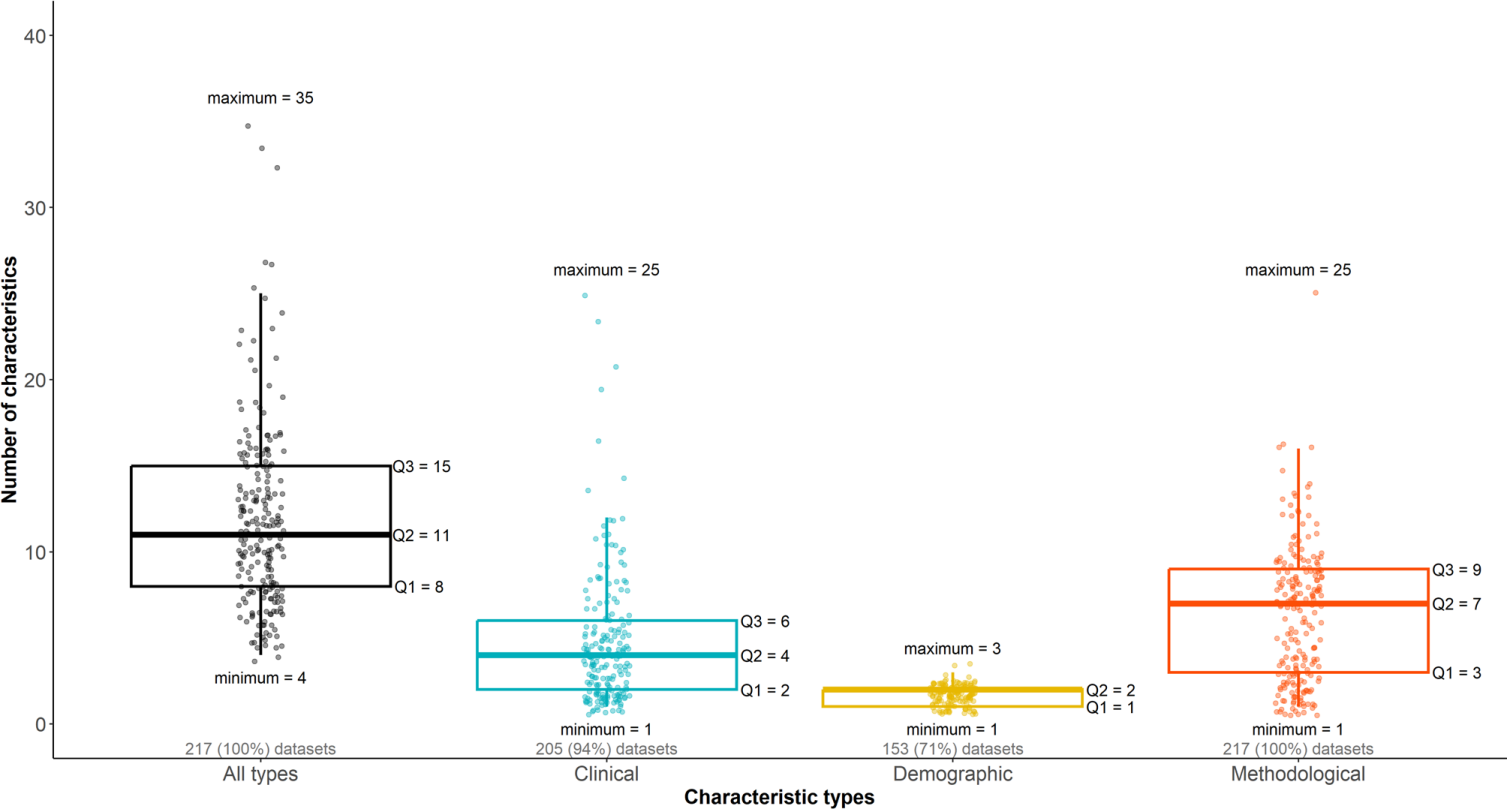
Box plots with integrated dots on the number of characteristics for all datasets and per characteristic type. Five quartiles accompany all box plots, including the minimum, the first quartile (Q1), the median (Q2), the third quartile (Q3), and the maximum. The number and percentage of corresponding datasets (out of 217) appear in grey below the box plots

Figure 1 presents the distribution of the number of characteristics across all datasets, as well as based on the characteristic type. Overall, the number of characteristics ranged from 4 to 35 (median: 11), with the middle half comprising 8 to 15 characteristics (black box plot). There was a wide range of clinical and methodological characteristics, each spanning from 1 to 25, with the middle half of the methodological characteristics being slightly higher (IQR: 3–9) than the clinical characteristics (IQR: 2–6) (Fig. 1). Most datasets (88%, $\:n=191$) comprised 5 to 17 characteristics, while very few datasets included more characteristics, with one containing 35 characteristics (Additional file 3: Figure S1); a similar pattern was observed regarding the clinical and methodological characteristics.

**Fig. 2:**
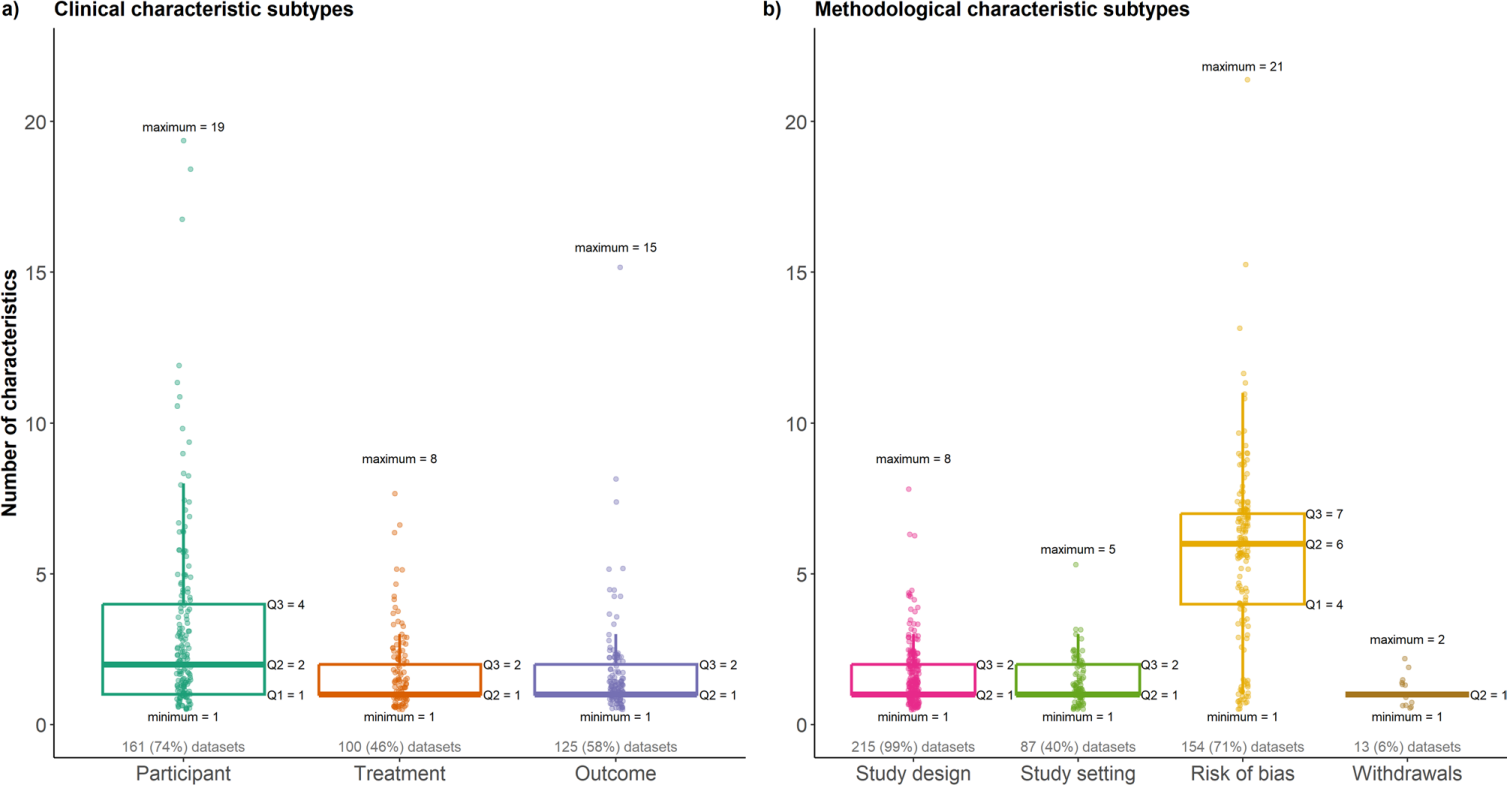
Box plots with integrated dots on the number of characteristics for each clinical characteristic subtype (plot a)) and each methodological characteristic type (plot b)). Five quartiles accompany all box plots, including the minimum, the first quartile (Q1), the median (Q2), the third quartile (Q3), and the maximum. The number and percentage of corresponding datasets (out of 217) appear in grey below the box plots

Figure 2 illustrates the distribution of the number of characteristics when distinguishing between clinical and methodological subtypes. Among the clinical characteristics (Fig. 2a), participant-related ones had the widest range, spanning from 1 to 19 characteristics; however, the middle half of these characteristics comprised a notably smaller number (IQR: 1–4). Treatment and outcome characteristics had a similar distribution overall, with a median equal to 1 and overlapping narrow IQR of 1 to 2 characteristics. Regarding methodological characteristic subtypes (Fig. 2b), the risk of bias stood out for having the widest range (1 to 21 characteristics) and IQR (4 to 7 characteristics) compared to the other subtypes that ranged from 1 to 8 characteristics and had very narrow IQR of 1 to 2 characteristics.

**Fig. 3:**
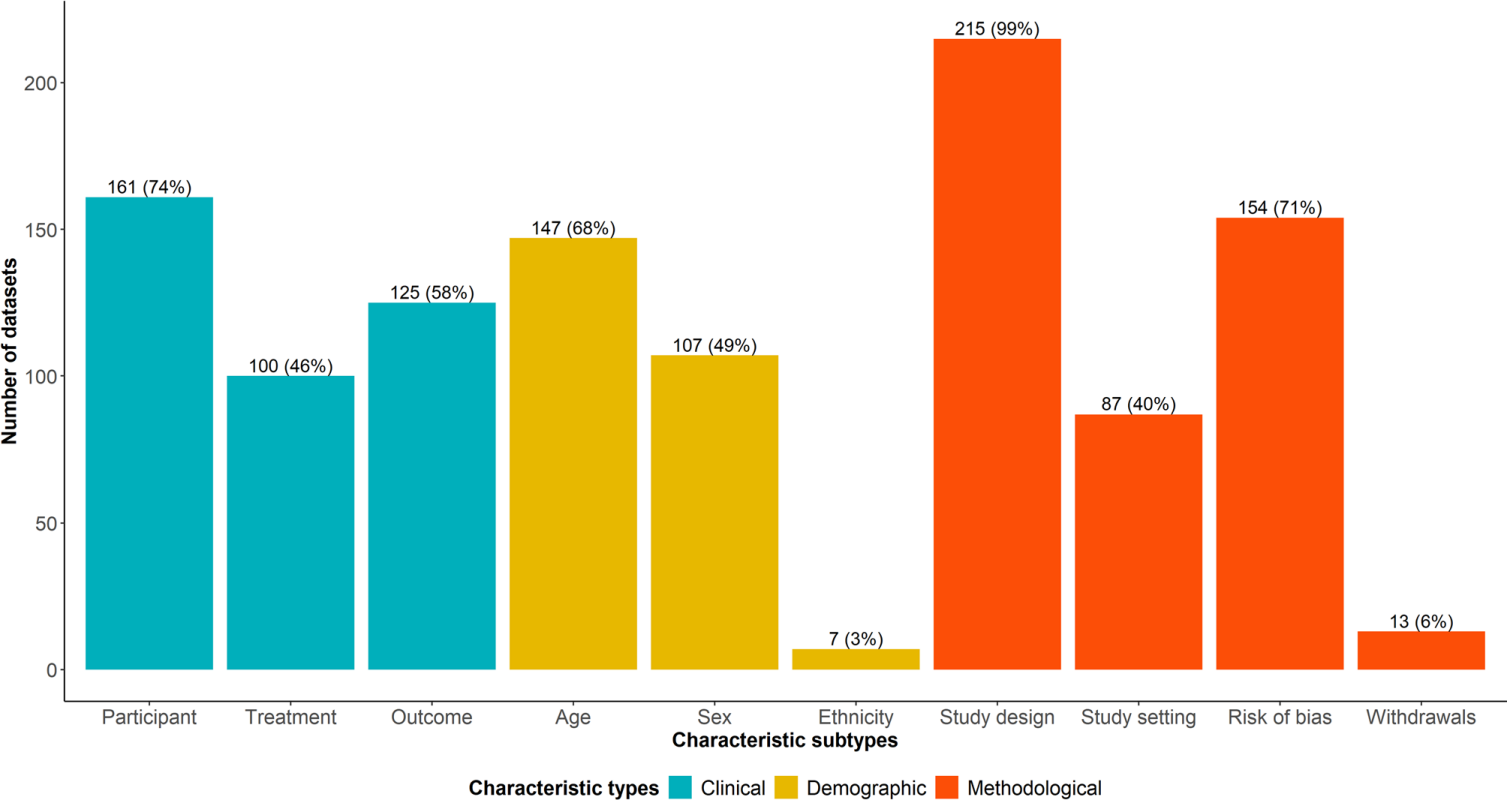
Grouped bar plots on the number and percentage of datasets that include at least one characteristic for the different characteristic subtypes. Different colours are used to indicate the different characteristic types. Percentages are calculated out of 217 datasets

All datasets contained at least one methodological characteristic, with study design being the dominant subtype (99%, $\:n=215$), followed by risk of bias (71%, $\:n=154$) and study setting (40%, $\:n=87$) (Fig. 3). Clinical characteristics comprised the second dominant characteristic type, with 74% ($\:n=161$) of the datasets including at least one participant characteristic, followed by outcome (58%, $\:n=125$) and treatment characteristics (46%, $\:n=100$) (Fig. 3). Age was the most frequently reported demographic characteristic (68%, $\:n=147$), and ethnicity was the least reported (3%, $\:n=7$).

#### Commonness of combinations of the characteristic types and subtypes

**Fig. 4:**
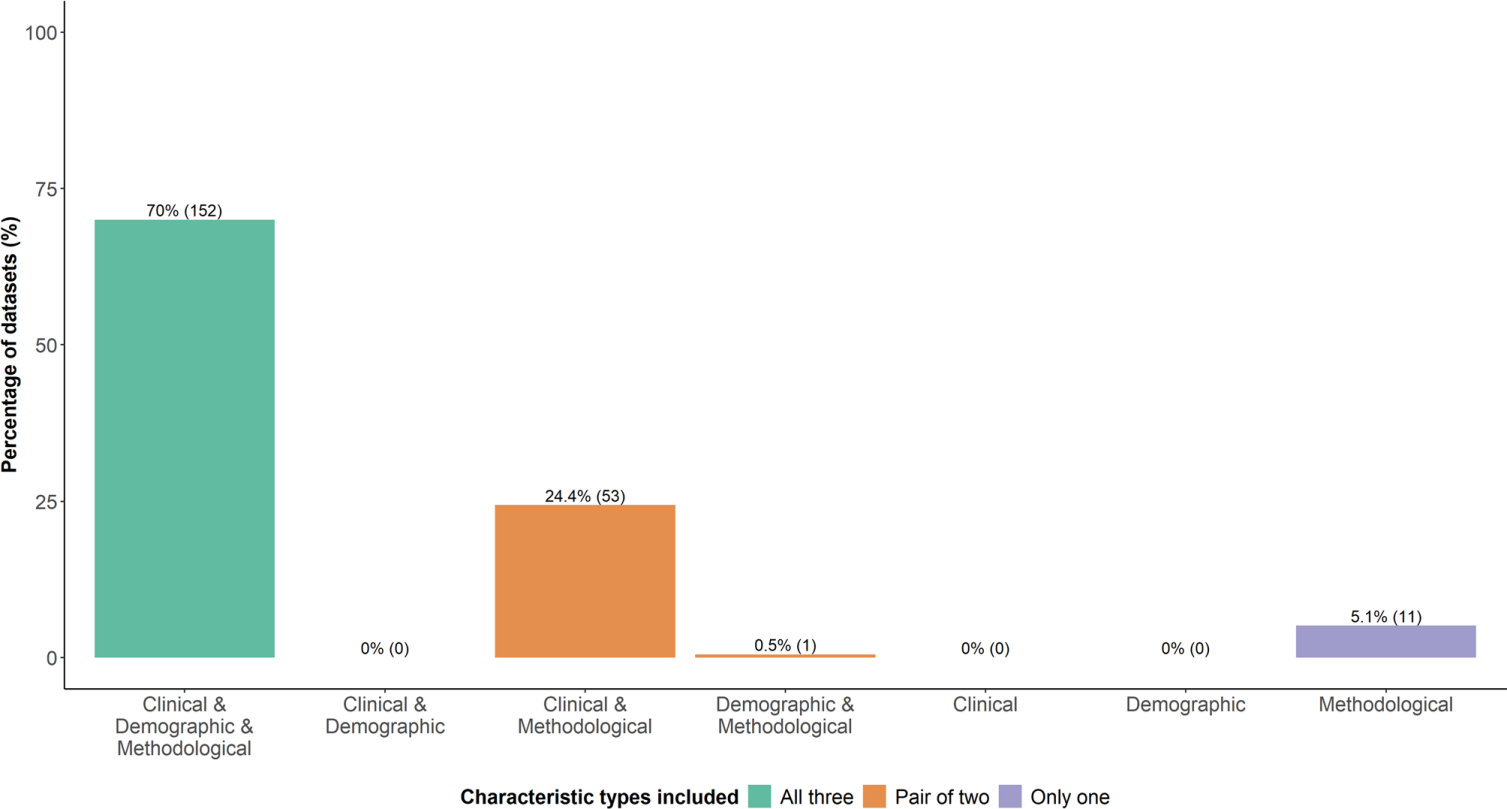
Grouped bar plots on the number and percentage of datasets that include the different combinations of clinical, demographic and methodological characteristics. The x-axis refers to the different combinations. Different colours are used to indicate the number of types in each combination. Percentages are calculated out of 217 datasets

Most datasets were populated with all three characteristic types (70%,$\:\:n=152$), followed by 53 (24%) datasets that included only clinical and methodological characteristics, 11 (5%) datasets with only methodological characteristics, and one dataset comprising demographic and methodological characteristics (Fig. 4). None of the datasets included only clinical or demographic characteristics or their combination.

**Fig. 5:**
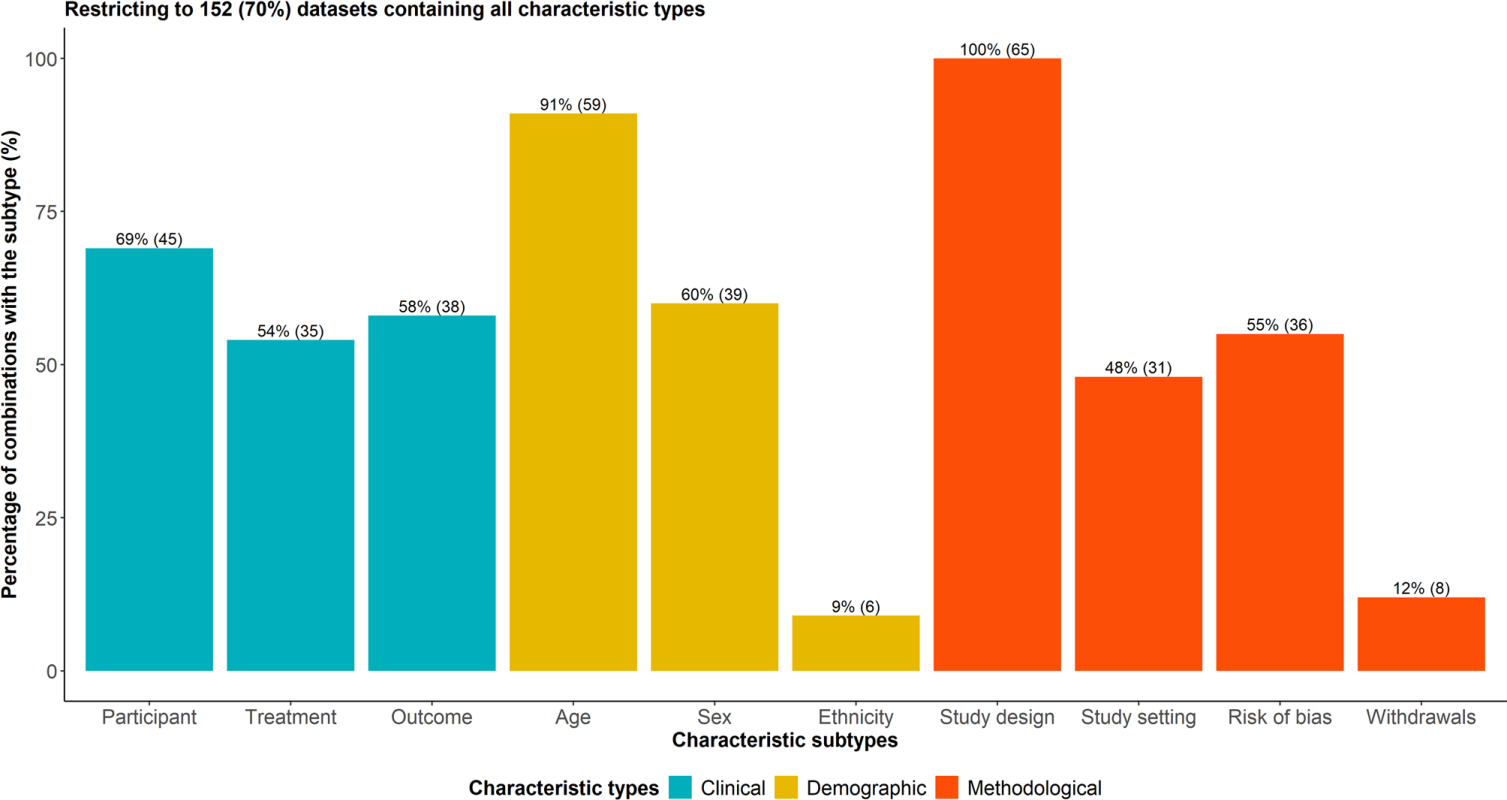
Grouped bar plots on the percentage of combinations of characteristic types that include each characteristic subtype. Only 152 datasets were considered that contained all characteristic types. Different colours are used to indicate the different characteristic types. Percentages are calculated out of 152 datasets

Sixty-five combinations among the ten characteristic subtypes were detected in the 152 datasets containing all three characteristic types. Of those, 37 (57%) combinations appeared only once. The frequency of the remaining 28 combinations ranged from 2 to 14, with a median of 3 (IQR: 2–5). The most frequent combination, appearing in 14 datasets, included participant and outcome characteristics, age, sex, study design information and risk of bias domains. None of the combinations included all ten characteristic subtypes. Study design features populated all 65 combinations, followed by age (91%, $\:n=59$) and participant characteristics (69%, $\:n=45$) (Fig. 5). Ethnicity (9%, $\:n=6$) and withdrawals (12%, $\:n=8)$ appeared the least in the combinations.

### Describing the distribution and commonness of missing data

**Fig. 6:**
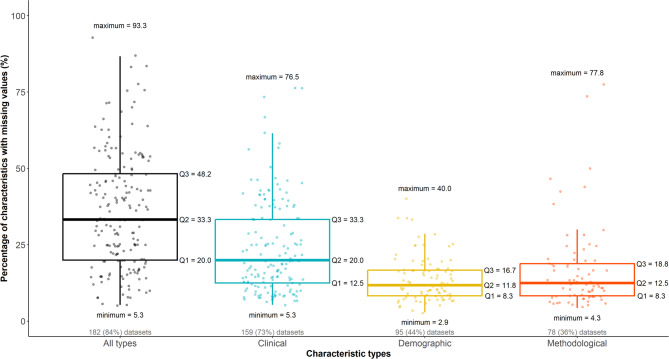
Box plots with integrated dots on the percentage of characteristics with missing data across all datasets and per characteristic type. Five quartiles accompany all box plots, including the minimum, the first quartile (Q1), the median (Q2), the third quartile (Q3), and the maximum. The number and percentage of corresponding datasets (out of 217) with missing data appear in grey below the box plots

The percentage of total missing data varied notably across the datasets, ranging from 0.2 to 32.9% (median: 7%, IQR: 2.5–12.5%). Of the 217 datasets, 182 (84%) contained at least one characteristic with missing data. Figure 6 illustrates the distribution of the percentage of characteristics with at least one missing data across all datasets (black box plot) and by characteristic type. These characteristics ranged from 1 to 22 across the datasets (median: 4, IQR: 2–7), corresponding to a range of 5.3–93.3% of the included characteristics (median: 33.3%, IQR: 20.0–48.2%) (Fig. 6). Clinical characteristics with missing data populated most datasets (73%, $\:n=159$), followed by demographic and methodological characteristics that contained missing data in 95 (44%) and 78 (36%) datasets, respectively (Fig. 6). Furthermore, clinical characteristics exhibited the highest variability in the percentage of characteristics with missing data, ranging from 5.3 to 76.5% (median: 20.0%, IQR: 12.5–33.3%), followed by methodological characteristics (median: 12.5%, IQR: 8.3–18.8%), and demographics characteristics (median: 11.8%, IQR: 8.3–16.7%) (Fig. 6).

When differentiating among the clinical subtypes, participant characteristics with missing data occupied more than half the datasets (56%, $\:n=122$), followed by outcome and treatment characteristics containing missing data in 24% ($\:n=52$) and 21% ($\:n=45$) of the datasets, respectively (Additional file 3: Figure S2a). In addition, participant characteristics were associated with greater variability in the percentage of characteristics with missing data, ranging from 5.0 to 73.3% (median: 18.2%, IQR: 10.3–30.8%), with the treatment and outcome subtypes sharing the same midspread of percentage characteristics with missing data at lower values (median: 10.0%, IQR: 7.7–14.3%). On the contrary, methodological subtypes with missing data were less common, found in 5–15% of the datasets (Additional file 3: Figure S2b). Among the methodological subtypes, the risk of bias domains displayed the highest dispersion in the percentage of characteristics with missing data, ranging from 4.3 to 77.8% (median: 11.2%, IQR: 6.2–25.7%), and withdrawals exhibited the least dispersion and at lower values, ranging from 3.7 to 16.7% (median: 8.7%, IQR: 7.8–10.0%) (Additional file 3: Figure S2b).

## Discussion

The present article has introduced the tracenma R package [21] that hosts a database of 217 connected networks with aggregate study-level characteristics extracted from the respective systematic reviews with multiple treatments. Tracenma [21] is the first database built to allow assessing new methodological advances in transitivity evaluation using empirical data. The database encompasses networks of various geometries and information concerning the number of studies, treatments, and sparsity of observed comparisons, as well as numerous extracted characteristics related to the design and target population of the included studies from several investigated healthcare fields.

The nmadb database [13] comprised the pillar of tracenma [21], above which a database of connected networks was constructed and facilitated extraction by providing the studies and treatment comparisons forming the connected networks. We consulted the relevant literature to determine the necessary characteristics constituting potential effect modifiers [31–34]. We distinguished the characteristics into three major types and subsequent subtypes to investigate their distribution in the database and identify underrepresented characteristics. We found that demographic characteristics were the least frequently reported despite having a central role in determining the eligibility criteria. On the contrary, methodological characteristics dominated all datasets in tracenma [21] and were less prone to missing data. A possible explanation may be that methodological characteristics, such as study design and risk of bias, are commonly defined across all healthcare fields, diminishing the risk of being underreported in systematic reviews. However, not all methodological subtypes received the same attention: study design was ubiquitous in the datasets, while withdrawals were hardly reported.

Clinical characteristics were present in most datasets and were subject to most missing data. Contrary to methodological characteristics, clinical characteristics are tailored to the research question, broadness of eligibility criteria, target population and treatments, resulting in great variability in their number and content even across studies investigating the same condition, and hence, a heterogeneous external validity [40]. Furthermore, the expertise of the clinicians and review authors on the investigated clinical topic greatly affects the selected effect modifiers, implicating the reporting completeness of the characteristics at the study and systematic review level [40, 41]. We found that the clinical characteristic subtypes received uneven attention, with participant characteristics being reported more frequently. Treatment characteristics were the least extractable subtype for being often reported as a mix of numbers and text, making this subtype appear spuriously underrepresented in the database.

Seventy per cent of the datasets included all three characteristic types; however, the types and subtypes varied substantially in frequency, indicating a lack of comprehensive reporting on the relevant characteristics in these datasets, which may limit the quality of the transitivity assessment. Even when all three types are present, individual datasets may still be incomplete, as the specific subtypes and their relevance to the research question can vary considerably. While systematic reviews are transparent regarding the effect modifiers considered, they tend not to report the decision-making process for selecting them [42]. There is also no empirical evidence on the relevance and completeness (for transitivity assessment) of the selected effect modifiers in systematic reviews, which would have allowed for gauging the quality of transitivity evaluation: systematically missing or irrelevant effect modifiers would prevent a thorough and reliable evaluation of transitivity, with implications for the credibility of the conclusion from the NMA results. Such an empirical study would require specifying the healthcare fields and conditions to provide targeted evidence and recommendations in selecting the necessary effect modifiers to corroborate an in-depth transitivity evaluation.

The present study has limitations that require acknowledgement and are also relevant to the tracenma database [21]. The reliance on the nmadb database [13] limits the comprehensiveness of the tracenma database [21]. The nmadb database [13] only includes systematic reviews published from 1999 to 2015. Newer systematic reviews, or those outside the nmadb database [13], are not considered, missing much of the relevant, available evidence. It is unclear whether more recent systematic reviews would contain more extractable characteristics. Furthermore, the geographic and clinical scope of the included networks is not analysed. If the tracenma database [21] primarily consists of studies from high-income countries or specific medical fields, the findings by applying a transitivity evaluation method (for instance [29]), may not be generalisable to all NMAs. We did not judge the relevance and completeness (for transitivity evaluation) of the extracted characteristics found in the systematic reviews, as it would require clinical expertise. For instance, ethnicity was rare in the tracenma database [21], raising questions about the completeness of the corresponding datasets’ characteristics and potentially limiting the usability of these datasets. Limitations in the relevance and completeness of the extracted characteristics found in the datasets of the tracenma database [21] would also reflect limitations in the PICO features defined in the corresponding systematic reviews. Therefore, the tracenma database [21] is unsuitable for empirically mapping the reported effect modifiers in different healthcare settings or making generalisations about the commonness of transitivity in a specific healthcare setting using a transitivity assessment method.

There were missing data in several datasets of the tracenma database [21]. We did not attempt to impute missing data, as the tracenma database [21] aimed to capture common reporting limitations encountered in published systematic reviews, including heterogeneity in the reported characteristics, leading to missing data in some studies. Addressing missing data in the context of transitivity assessment is out of the scope of the present study and requires immediate methodological attention. The tracenma database [21] can be used to explore the implications of imputing or excluding missing data on the results’ robustness based on a method for transitivity assessment. For instance, if certain characteristics are frequently missing, how does this affect transitivity evaluation? If missing data are excluded, does this create a bias toward certain studies?

The tracenma database [21] includes only systematic reviews that provided at least four extractable characteristics in their report. Even though technically, we could evaluate transitivity with as few characteristics as two or three, we would have missed many critical characteristic types and subtypes necessary to ensure a comprehensive and reliable transitivity evaluation. Eventually, one may also criticise our threshold of four characteristics as still missing several characteristic types and subtypes and that we should have considered a higher threshold; however, our ultimate goal was to create a database with as many systematic reviews as technically possible that also reflects the limitations of the available evidence base, such as missing data and underreporting of some characteristic types and subtypes. To our knowledge, there is no universal consensus on what constitutes a methodologically sound number of characteristics to allow for a comprehensive evaluation of the transitivity assumption. From our point of view, such a threshold would be challenging to define (theoretically, statistically or empirically), as the number of characteristics included in a systematic review *also* depends on the reporting quality of the included studies and, by extension, on the willingness of the review authors to request the missing characteristics and the study authors to respond promptly.

The extraction challenges outlined in the Methods section are also limitations of our study. We attempted to retain many of the characteristics and studies in the datasets by addressing the extraction challenges carefully; however, the assumptions made were often strong (e.g., assuming the mean and median were the same), potentially compromising the extraction accuracy to an unknown extent. We did not contact the authors of systematic reviews to clarify typing errors and missing information during the extraction.

These limitations highlight the need for careful consideration of data quality and potential biases when developing and applying transitivity assessment methods. Future research in the methodology for transitivity evaluation should prioritise (1) developing methods robust to missing data and variations in reporting quality, (2) exploring the sensitivity of transitivity assessments to the assumptions made during data extraction, and (3) establishing guidelines for reporting characteristics relevant to transitivity assessment in systematic reviews.

## Conclusions

We created a database with 217 datasets containing study-level aggregate characteristics that are potential effect modifiers. The database is hosted in the tracenma R package [21] with functions that facilitate data access. Directions and examples on navigating the tracenma R package [21] can be found on the repository’s website [39]. The methodological gap in assessing the transitivity assumption motivated the development of the tracenma R package [21], aiming to initiate methodological advances in transitivity assessment using empirical data. Prioritising research around transitivity assessment, including suitable statistical methods and a framework to define the important effect modifiers underpinning the target condition, deserves the immediate attention of the broad evidence synthesis community of statisticians, methodologists and clinicians.

## Supplementary Information

Additional file 1. Excluded networks. List of excluded networks grouped by reason for exclusion

Additional file 2. Illustrating transitivity evaluation with tracenma

Additional file 3. Distribution of the number of characteristics across datasets in the tracenma database. Figure S2. Box plots with integrated dots on the percentage of characteristics with missing data across all datasets, distinguishing among the clinical and methodological characteristic subtypes

## Data Availability

The data supporting the present study’s findings can be found in the tracenma R package [21]. The functions related to the present study to reproduce the results are publicly available at https://github.com/LoukiaSpin/Transitivity-evaluation-tracenma-description.git.

## Abbreviations

IQR: Interquartile range
NMA: Network meta-analysis
