# Supplementary material for: A database to initiate methodological advances in the evaluation of transitivity assumption in network meta-analysis: qualitative features and limitations of the tracenma R package
Source: BMC Med Res Methodol. 2025 Jul 31;25:183. doi: 10.1186/s12874-025-02634-x (PMC12315375; doi:10.1186/s12874-025-02634-x)
Supplement: Supplementary file 1 — Additional file 1. Excluded networks. List of excluded networks grouped by reason for exclusion [file 12874_2025_2634_MOESM1_ESM.docx]

**Supporting Information for the article 'A database to initiate methodological advances in the evaluation of transitivity assumption in network meta-analysis: qualitative features and limitations of the tracenma R package'**

Loukia M. Spineli^1^  [Spineli.Loukia@mh-hannover.de](mailto:Spineli.Loukia@mh-hannover.de)

Andrés Mauricio García-Sierra^2^ [andresmauriciog@uchicago.edu](mailto:andresmauriciog@uchicago.edu)

Juan Jose Yepes-Nuñez^3,4^  [jj.yepesn@uniandes.edu.co](mailto:jj.yepesn@uniandes.edu.co)

^1^Midwifery Research and Education Unit, Hannover Medical School, Hannover, Germany

^2^Department of Public Health Sciences, University of Chicago, Chicago, USA

^3^School of Medicine, Universidad de los Andes, Bogotá D.C., Colombia

^4^Internal Medicine Department, Fundación Santa Fe de Bogotá, Bogotá D.C., Colombia

**List of excluded networks grouped by reasons for exclusion**

*Networks were not extracted (outcome data unavailable)*

1. Djavan B, Marberger M. A meta-analysis on the efficacy and tolerability of alpha1-adrenoceptor antagonists in patients with lower urinary tract symptoms suggestive of benign prostatic obstruction. *Eur Urol* 1999;36(1):1-13.
2. Wilhelmus KR. The treatment of herpes simplex virus epithelial keratitis. *Trans Am Ophthalmol Soc* 2000;98:505-32.
3. Wehren LE, Hosking D, Hochberg MC. Putting evidence-based medicine into clinical practice: comparing anti-resorptive agents for the treatment of osteoporosis. *Curr Med Res Opin* 2004;20(4):525-31.
4. Brophy JM, Joseph L. Medical decision making with incomplete evidence--choosing a platelet glycoprotein IIbIIIa receptor inhibitor for percutaneous coronary interventions. *Med Decis Making* 2005;25(2):222-8.
5. Richy F, Schacht E, Bruyere O, Ethgen O, Gourlay M, Reginster JY. Vitamin D analogs versus native vitamin D in preventing bone loss and osteoporosis-related fractures: a comparative meta-analysis. *Calcif Tissue Int* 2005;76(3):176-86.
6. Davies L, Brown TJ, Haynes S, Payne K, Elliott RA, McCollum C. Cost-effectiveness of cell salvage and alternative methods of minimising perioperative allogeneic blood transfusion: a systematic review and economic model. *Health Technol Assess* 2006;10(44):iii-iv, ix-x, 1-210.
7. Eckert L, Lançon C. Duloxetine compared with fluoxetine and venlafaxine: use of meta-regression analysis for indirect comparisons. *BMC Psychiatry* 2006;6:30.
8. Gartlehner G, Hansen RA, Jonas BL, Thieda P, Lohr KN. The comparative efficacy and safety of biologics for the treatment of rheumatoid arthritis: a systematic review and metaanalysis. *J Rheumatol* 2006;33(12):2398-408.
9. Kyrgiou M, Salanti G, Pavlidis N, Paraskevaidis E, Ioannidis JP. Survival benefits with diverse chemotherapy regimens for ovarian cancer: meta-analysis of multiple treatments. *J Natl Cancer Inst* 2006;98(22):1655-63.
10. Wu P, Wilson K, Dimoulas P, Mills EJ. Effectiveness of smoking cessation therapies: a systematic review and meta-analysis. *BMC Public Health* 2006;6:300.
11. Buscemi N, Vandermeer B, Friesen C, Bialy L, Tubman M, Ospina M, Klassen TP, Witmans M. The efficacy and safety of drug treatments for chronic insomnia in adults: a meta-analysis of RCTs. *J Gen Intern Med* 2007;22(9):1335-50.
12. Golfinopoulos V, Salanti G, Pavlidis N, Ioannidis JP. Survival and disease-progression benefits with treatment regimens for advanced colorectal cancer: a meta-analysis. *Lancet Oncol* 2007;8(10):898-911.
13. Tudur Smith C, Marson AG, Chadwick DW, Williamson PR. Multiple treatment comparisons in epilepsy monotherapy trials. *Trials* 2007;8:34.
14. Hansen RA, Gaynes BN, Gartlehner G, Moore CG, Tiwari R, Lohr KN. Efficacy and tolerability of second-generation antidepressants in social anxiety disorder*. Int Clin Psychopharmacol* 2008;23(3):170-9.
15. Hofmeyr GJ, Gülmezoglu AM. Misoprostol for the prevention and treatment of postpartum haemorrhage. *Best Pract Res Clin Obstet Gynaecol* 2008;22(6):1025-41.
16. Mauri D, Polyzos NP, Salanti G, Pavlidis N, Ioannidis JP. Multiple-treatments meta-analysis of chemotherapy and targeted therapies in advanced breast cancer. *J Natl Cancer Inst* 2008;100(24):1780-91.
17. Kotzé A, Scally A, Howell S. Efficacy and safety of different techniques of paravertebral block for analgesia after thoracotomy: a systematic review and metaregression. *Br J Anaesth* 2009;103(5):626-36.
18. Edwards SJ, Smith CJ. Tolerability of atypical antipsychotics in the treatment of adults with schizophrenia or bipolar disorder: a mixed treatment comparison of randomized controlled trials. *Clin Ther* 2009;31 Pt 1:1345-59.
19. Hawkins N, Scott DA, Woods BS, Thatcher N. No study left behind: a network meta-analysis in non-small-cell lung cancer demonstrating the importance of considering all relevant data. *Value Health* 2009;12(6):996-1003.
20. Quilici S, Chancellor J, Löthgren M, Simon D, Said G, Le TK, Garcia-Cebrian A, Monz B. Meta-analysis of duloxetine vs. pregabalin and gabapentin in the treatment of diabetic peripheral neuropathic pain. *BMC Neurol* 2009;9:6.
21. Delahoy P, Thompson S, Marschner IC. Pregabalin versus gabapentin in partial epilepsy: a meta-analysis of dose-response relationships. *BMC Neurol* 2010;10:104.
22. Häuser W, Petzke F, Sommer C. Comparative efficacy and harms of duloxetine, milnacipran, and pregabalin in fibromyalgia syndrome. *J Pain* 2010;11(6):505-21.
23. Häuser W, Petzke F, Üçeyler N, Sommer C. Comparative efficacy and acceptability of amitriptyline, duloxetine and milnacipran in fibromyalgia syndrome: a systematic review with meta-analysis. *Rheumatology (Oxford)* 2011;50(3):532-43.
24. Orme M, Collins S, Dakin H, Kelly S, Loftus J. Mixed treatment comparison and meta-regression of the efficacy and safety of prostaglandin analogues and comparators for primary open-angle glaucoma and ocular hypertension. *Curr Med Res Opin* 2010;26(3):511-28.
25. Piscione F, Piccolo R, Cassese S, Galasso G, De Rosa R, D'Andrea C, Chiariello M. Effect of drug-eluting stents in patients with acute ST-segment elevation myocardial infarction undergoing percutaneous coronary intervention: a meta-analysis of randomised trials and an adjusted indirect comparison. EuroIntervention. 2010 Feb;5(7):853-60.
26. Tu YK, Woolston A, Faggion CM Jr. Do bone grafts or barrier membranes provide additional treatment effects for infrabony lesions treated with enamel matrix derivatives? A network meta-analysis of randomized-controlled trials. *J Clin Periodontol* 2010;37(1):59-79.
27. Vissers D, Stam W, Nolte T, Lenre M, Jansen J. Efficacy of intranasal fentanyl spray versus other opioids for breakthrough pain in cancer. *Curr Med Res Opin* 2010;26(5):1037-45.
28. Baldwin D, Woods R, Lawson R, Taylor D. Efficacy of drug treatments for generalised anxiety disorder: systematic review and meta-analysis. *BMJ* 2011;342:d1199.
29. Bekkering GE, Soares-Weiser K, Reid K, Kessels AG, Dahan A, Treede RD, Kleijnen J. Can morphine still be considered to be the standard for treating chronic pain? A systematic review including pair-wise and network meta-analyses. *Curr Med Res Opin* 2011;27(7):1477-91.
30. Dakin HA, Welton NJ, Ades AE, Collins S, Orme M, Kelly S. Mixed treatment comparison of repeated measurements of a continuous endpoint: an example using topical treatments for primary open-angle glaucoma and ocular hypertension. *Stat Med* 2011;30(20):2511-35.
31. Danchin N, Marzilli M, Parkhomenko A, Ribeiro JP. Efficacy comparison of trimetazidine with therapeutic alternatives in stable angina pectoris: a network meta-analysis. *Cardiology* 2011;120(2):59-72.
32. Choy E, Marshall D, Gabriel ZL, Mitchell SA, Gylee E, Dakin HA. A systematic review and mixed treatment comparison of the efficacy of pharmacological treatments for fibromyalgia. *Semin Arthritis Rheum* 2011;41(3):335-45.e6.
33. Freemantle N, Lafuente-Lafuente C, Mitchell S, Eckert L, Reynolds M. Mixed treatment comparison of dronedarone, amiodarone, sotalol, flecainide, and propafenone, for the management of atrial fibrillation. *Europace* 2011;13(3):329-45.
34. Freemantle N, Tharmanathan P, Herbrecht R. Systematic review and mixed treatment comparison of randomized evidence for empirical, pre-emptive and directed treatment strategies for invasive mould disease. *J Antimicrob Chemother* 2011;66 Suppl 1:i25-35.
35. Gross JL, Kramer CK, Leitão CB, Hawkins N, Viana LV, Schaan BD, Pinto LC, Rodrigues TC, Azevedo MJ; Diabetes and Endocrinology Meta-analysis Group (DEMA). Effect of antihyperglycemic agents added to metformin and a sulfonylurea on glycemic control and weight gain in type 2 diabetes: a network meta-analysis. *Ann Intern Med* 2011;154(10):672-9.
36. Kessler TM, Bachmann LM, Minder C, Löhrer D, Umbehr M, Schünemann HJ, Kessels AG. Adverse event assessment of antimuscarinics for treating overactive bladder: a network meta-analytic approach. *PLoS One* 2011;6(2):e16718.
37. McIntosh B, Cameron C, Singh SR, Yu C, Ahuja T, Welton NJ, Dahl M. Second-line therapy in patients with type 2 diabetes inadequately controlled with metformin monotherapy: a systematic review and mixed-treatment comparison meta-analysis. *Open Med* 2011;5(1):e35-48.
38. Mills EJ, Druyts E, Ghement I, Puhan MA. Pharmacotherapies for chronic obstructive pulmonary disease: a multiple treatment comparison meta-analysis. *Clin Epidemiol* 2011;3:107-29.
39. Pichenot M, Deuffic-Burban S, Cuzin L, Yazdanpanah Y. Efficacy of new antiretroviral drugs in treatment-experienced HIV-infected patients: a systematic review and meta-analysis of recent randomized controlled trials. *HIV Med* 2012;13(3):148-55.
40. Riemsma R, Forbes C, Harker J, Worthy G, Misso K, Schäfer M, Kleijnen J, Stürzebecher S. Systematic review of tapentadol in chronic severe pain. *Curr Med Res Opin* 2011;27(10):1907-30.
41. Selph S, Carson S, Fu R, Thakurta S, Low A, McDonagh M. Drug Class Review: Neuropathic Pain: Final Update 1 Report [Internet]. Portland (OR): Oregon Health & Science University; 2011.
42. Smith B, Peterson K, Fu R, McDonagh M, Thakurta S. Drug Class Review: Drugs for Fibromyalgia: Final Original Report [Internet]. Portland (OR): Oregon Health & Science University; 2011.
43. Trelle S, Reichenbach S, Wandel S, Hildebrand P, Tschannen B, Villiger PM, Egger M, Jüni P. Cardiovascular safety of non-steroidal anti-inflammatory drugs: network meta-analysis. *BMJ* 2011;342:c7086.
44. Wiebe N, Padwal R, Field C, Marks S, Jacobs R, Tonelli M. A systematic review on the effect of sweeteners on glycemic response and clinically relevant outcomes. *BMC Med* 2011;9:123.
45. Ziogas DC, Voulgarelis M, Zintzaras E. A network meta-analysis of randomized controlled trials of induction treatments in acute myeloid leukemia in the elderly. *Clin Ther* 2011;33(3):254-79.
46. Alberton M, Wu P, Druyts E, Briel M, Mills EJ. Adverse events associated with individual statin treatments for cardiovascular disease: an indirect comparison meta-analysis. *QJM* 2012;105(2):145-57.
47. Asseburg C, Peura P, Oksanen T, Turunen J, Purmonen T, Martikainen J. Cost-effectiveness of oral triptans for acute migraine: mixed treatment comparison. *Int J Technol Assess Health Care* 2012;28(4):382-9.
48. Bangalore S, Kumar S, Fusaro M, Amoroso N, Attubato MJ, Feit F, Bhatt DL, Slater J. Short- and long-term outcomes with drug-eluting and bare-metal coronary stents: a mixed-treatment comparison analysis of 117 762 patient-years of follow-up from randomized trials. *Circulation* 2012;125(23):2873-91.
49. Bangalore S, Kumar S, Fusaro M, Amoroso N, Kirtane AJ, Byrne RA, Williams DO, Slater J, Cutlip DE, Feit F. Outcomes with various drug eluting or bare metal stents in patients with diabetes mellitus: mixed treatment comparison analysis of 22,844 patient years of follow-up from randomised trials. *BMJ* 2012;345:e5170.
50. Buser N, Ivic S, Kessler TM, Kessels AG, Bachmann LM. Efficacy and adverse events of antimuscarinics for treating overactive bladder: network meta-analyses. *Eur Urol* 2012;62(6):1040-60.
51. Cohen A, Drost P, Marchant N, Mitchell S, Orme M, Rublee D, Simon TA, Sutton A. The efficacy and safety of pharmacological prophylaxis of venous thromboembolism following elective knee or hip replacement: systematic review and network meta-analysis. *Clin Appl Thromb Hemost* 2012;18(6):611-27.
52. Cooper C, Lester R, Thorlund K, Druyts E, El Khoury AC, Yaya S, Mills EJ. Direct-acting antiviral therapies for hepatitis C genotype 1 infection: a multiple treatment comparison meta-analysis. *QJM* 2013;106(2):153-63.
53. Dequen P, Lorigan P, Jansen JP, van Baardewijk M, Ouwens MJ, Kotapati S. Systematic review and network meta-analysis of overall survival comparing 3 mg/kg ipilimumab with alternative therapies in the management of pretreated patients with unresectable stage III or IV melanoma. *Oncologist* 2012;17(11):1376-85.
54. Freemantle N, Cooper C, Diez-Perez A, Gitlin M, Radcliffe H, Shepherd S, Roux C. Results of indirect and mixed treatment comparison of fracture efficacy for osteoporosis treatments: a meta-analysis. *Osteoporos Int* 2013;24(1):209-17.
55. Harenberg J, Marx S, Dahl OE, Marder VJ, Schulze A, Wehling M, Weiss C. Interpretation of endpoints in a network meta-analysis of new oral anticoagulants following total hip or total knee replacement surgery. *Thromb Haemost* 2012;108(5):903-12.
56. Hua et al. A meta-analysis of infantile rotavirus enteritis interventions. World Journal of Gastroenterology. 2012. nmadb ID: 481643.
57. Martyn-St James M, Glanville J, McCool R, Duffy S, Cooper J, Hugel P, Lane PW. The efficacy and safety of retigabine and other adjunctive treatments for refractory partial epilepsy: a systematic review and indirect comparison. *Seizure* 2012;21(9):665-78.
58. Mills EJ, Wu P, Lockhart I, Thorlund K, Puhan M, Ebbert JO. Comparisons of high-dose and combination nicotine replacement therapy, varenicline, and bupropion for smoking cessation: a systematic review and multiple treatment meta-analysis. *Ann Med* 2012;44(6):588-97.
59. Nüesch E, Häuser W, Bernardy K, Barth J, Jüni P. Comparative efficacy of pharmacological and non-pharmacological interventions in fibromyalgia syndrome: network meta-analysis. *Ann Rheum Dis* 2013;72(6):955-62.
60. Palmerini T, Biondi-Zoccai G, Della Riva D, Stettler C, Sangiorgi D, D'Ascenzo F, Kimura T, Briguori C, Sabatè M, Kim HS, De Waha A, Kedhi E, Smits PC, Kaiser C, Sardella G, Marullo A, Kirtane AJ, Leon MB, Stone GW. Stent thrombosis with drug-eluting and bare-metal stents: evidence from a comprehensive network meta-analysis. *Lancet* 2012;379(9824):1393-402.
61. Schmitz S, Adams R, Walsh CD, Barry M, FitzGerald O. A mixed treatment comparison of the efficacy of anti-TNF agents in rheumatoid arthritis for methotrexate non-responders demonstrates differences between treatments: a Bayesian approach. *Ann Rheum Dis* 2012;71(2):225-30.
62. Steiner S, Moertl D, Chen L, Coyle D, Wells GA. Network meta-analysis of prasugrel, ticagrelor, high- and standard-dose clopidogrel in patients scheduled for percutaneous coronary interventions. *Thromb Haemost* 2012;108(2):318-27.
63. Sun F, Yu K, Yang Z, Wu S, Zhang Y, Shi L, Ji L, Zhan S. Impact of GLP-1 receptor agonists on major gastrointestinal disorders for type 2 diabetes mellitus: a mixed treatment comparison meta-analysis. *Exp Diabetes Res* 2012;2012:230624.
64. Sun F, Yu K, Wu S, Zhang Y, Yang Z, Shi L, Ji L, Zhan S. Cardiovascular safety and glycemic control of glucagon-like peptide-1 receptor agonists for type 2 diabetes mellitus: a pairwise and network meta-analysis. *Diabetes Res Clin Pract* 2012;98(3):386-95.
65. Tang DH, Malone DC. A network meta-analysis on the efficacy of serotonin type 3 receptor antagonists used in adults during the first 24 hours for postoperative nausea and vomiting prophylaxis. *Clin Ther* 2012;34(2):282-94.
66. Thorlund K, Druyts E, Aviña-Zubieta JA, Mills EJ. Anti-tumor necrosis factor (TNF) drugs for the treatment of psoriatic arthritis: an indirect comparison meta-analysis. *Biologics* 2012;6:417-27.
67. Naci H, Ioannidis JP. Comparative effectiveness of exercise and drug interventions on mortality outcomes: metaepidemiological study. *BMJ* 2013;347:f5577.
68. Liu Y, Yan R, Song A, Niu X, Cao C, Wei J, Dong X, Gao D. Aliskiren/amlodipine vs. aliskiren/hydrochlorothiazide in hypertension: indirect meta-analysis of trials comparing the two combinations vs. monotherapy. *Am J Hypertens* 2014;27(2):268-78.
69. Brown T, Pilkington G, Bagust A, Boland A, Oyee J, Tudur-Smith C, Blundell M, Lai M, Martin Saborido C, Greenhalgh J, Dundar Y, Dickson R. Clinical effectiveness and cost-effectiveness of first-line chemotherapy for adult patients with locally advanced or metastatic non-small cell lung cancer: a systematic review and economic evaluation. *Health Technol Assess* 2013;17(31):1-278.
70. Palmerini T, Biondi-Zoccai G, Della Riva D, Mariani A, Sabaté M, Valgimigli M, Frati G, Kedhi E, Smits PC, Kaiser C, Genereux P, Galatius S, Kirtane AJ, Stone GW. Clinical outcomes with drug-eluting and bare-metal stents in patients with ST-segment elevation myocardial infarction: evidence from a comprehensive network meta-analysis. *J Am Coll Cardiol* 2013;62(6):496-504.
71. Naci H, Brugts JJ, Fleurence R, Tsoi B, Toor H, Ades AE. Comparative benefits of statins in the primary and secondary prevention of major coronary events and all-cause mortality: a network meta-analysis of placebo-controlled and active-comparator trials. *Eur J Prev Cardiol* 2013;20(4):641-57.
72. Maman K, Aballea S, Nazir J, Desroziers K, Neine ME, Siddiqui E, Odeyemi I, Hakimi Z. Comparative efficacy and safety of medical treatments for the management of overactive bladder: a systematic literature review and mixed treatment comparison. *Eur Urol* 2014;65(4):755-65.
73. Wiens A, Lenzi L, Venson R, Correr CJ, Rotta I, Pedroso ML, Pontarolo R. Comparative efficacy of oral nucleoside or nucleotide analog monotherapy used in chronic hepatitis B: a mixed-treatment comparison meta-analysis. *Pharmacotherapy* 2013;33(2):144-51.
74. Naci H, Brugts JJ, Fleurence R, Ades AE. Dose-comparative effects of different statins on serum lipid levels: a network meta-analysis of 256,827 individuals in 181 randomized controlled trials. *Eur J Prev Cardiol* 2013;20(4):658-70.
75. Skoetz N, Trelle S, Rancea M, Haverkamp H, Diehl V, Engert A, Borchmann P. Effect of initial treatment strategy on survival of patients with advanced-stage Hodgkin's lymphoma: a systematic review and network meta-analysis. *Lancet Oncol* 2013;14(10):943-52.
76. Li L, Tian J, Tian H, Sun R, Wang Q, Yang K. The efficacy and safety of different kinds of laparoscopic cholecystectomy: a network meta analysis of 43 randomized controlled trials. *PLoS One* 2014;9(2):e90313.
77. Datto C, Hellmund R, Siddiqui MK. Efficacy and tolerability of naproxen/esomeprazole magnesium tablets compared with non-specific NSAIDs and COX-2 inhibitors: a systematic review and network analyses. *Open Access Rheumatol* 2013;5:1-19.
78. Rashiq S, Vandermeer B, Abou-Setta AM, Beaupre LA, Jones CA, Dryden DM. Efficacy of supplemental peripheral nerve blockade for hip fracture surgery: multiple treatment comparison. *Can J Anaesth* 2013;60(3):230-43.
79. Shu T, Chen GH, Rong L, Feng F, Yang B, Chen R, Wang J. Indirect comparison of anti-TNF-α agents for active ankylosing spondylitis: mixed treatment comparison of randomized controlled trials. *Clin Exp Rheumatol* 2013;31(5):717-22.
80. Bangalore S, Amoroso N, Fusaro M, Kumar S, Feit F. Outcomes with various drug-eluting or bare metal stents in patients with ST-segment-elevation myocardial infarction: a mixed treatment comparison analysis of trial level data from 34 068 patient-years of follow-up from randomized trials. *Circ Cardiovasc Interv* 2013;6(4):378-90.
81. Navarese EP, Tandjung K, Claessen B, Andreotti F, Kowalewski M, Kandzari DE, Kereiakes DJ, Waksman R, Mauri L, Meredith IT, Finn AV, Kim HS, Kubica J, Suryapranata H, Aprami TM, Di Pasquale G, von Birgelen C, Kedhi E. Safety and efficacy outcomes of first and second generation durable polymer drug eluting stents and biodegradable polymer biolimus eluting stents in clinical practice: comprehensive network meta-analysis. *BMJ* 2013;347:f6530.
82. Jonas DE, Cusack K, Forneris CA, Wilkins TM, Sonis J, Middleton JC, Feltner C, Meredith D, Cavanaugh J, Brownley KA, Olmsted KR, Greenblatt A, Weil A, Gaynes BN. Psychological and Pharmacological Treatments for Adults With Posttraumatic Stress Disorder (PTSD) [Internet]. Rockville (MD): Agency for Healthcare Research and Quality (US); 2013 Apr. Report No.: 13-EHC011-EF.
83. Barth J, Munder T, Gerger H, Nüesch E, Trelle S, Znoj H, Jüni P, Cuijpers P. Comparative efficacy of seven psychotherapeutic interventions for patients with depression: a network meta-analysis. *PLoS Med* 2013;10(5):e1001454.
84. Cahill K, Stevens S, Perera R, Lancaster T. Pharmacological interventions for smoking cessation: an overview and network meta-analysis. *Cochrane Database Syst Rev* 2013;2013(5):CD009329. doi: 10.1002/14651858.CD009329.pub2.
85. Cope S, Donohue JF, Jansen JP, Kraemer M, Capkun-Niggli G, Baldwin M, Buckley F, Ellis A, Jones P. Comparative efficacy of long-acting bronchodilators for COPD: a network meta-analysis. *Respir Res* 2013;14(1):100.
86. Corbett MS, Rice SJ, Madurasinghe V, Slack R, Fayter DA, Harden M, Sutton AJ, Macpherson H, Woolacott NF. Acupuncture and other physical treatments for the relief of pain due to osteoarthritis of the knee: network meta-analysis. *Osteoarthritis Cartilage* 2013;21(9):1290-8.
87. Delea TE, Amdahl J, Chit A, Amonkar MM. Cost-effectiveness of lapatinib plus letrozole in her2-positive, hormone receptor-positive metastatic breast cancer in Canada. *Curr Oncol* 2013;20(5):e371-87.
88. Désaméricq G, Schurhoff F, Meary A, Szöke A, Macquin-Mavier I, Bachoud-Lévi AC, Maison P. Long-term neurocognitive effects of antipsychotics in schizophrenia: a network meta-analysis. *Eur J Clin Pharmacol* 2014;70(2):127-34.
89. Fang Y, Ding Y, Guo Q, Xing J, Long Y, Zong Z. Radioiodine therapy for patients with differentiated thyroid cancer after thyroidectomy: direct comparison and network meta-analyses. *J Endocrinol Invest* 2013;36(10):896-902.
90. Goralczyk AD, Cameron S, Amanzada A. Treatment of chronic HCV genotype 1 infection with telaprevir: a Bayesian mixed treatment comparison of fixed-length and response-guided treatment regimens in treatment-naïve and -experienced patients. *BMC Gastroenterol* 2013;13:148.
91. Goring S, Hawkins N, Wygant G, Roudaut M, Townsend R, Wood I, Barnett AH. Dapagliflozin compared with other oral anti-diabetes treatments when added to metformin monotherapy: a systematic review and network meta-analysis. *Diabetes Obes Metab* 2014;16(5):433-42.
92. Hadjigeorgiou GM, Doxani C, Miligkos M, Ziakas P, Bakalos G, Papadimitriou D, Mprotsis T, Grigoriadis N, Zintzaras E. A network meta-analysis of randomized controlled trials for comparing the effectiveness and safety profile of treatments with marketing authorization for relapsing multiple sclerosis. *J Clin Pharm Ther* 2013;38(6):433-9.
93. Hoaglin DC, Filonenko A, Glickman ME, Wasiak R, Gidwani R. Use of mixed-treatment-comparison methods in estimating efficacy of treatments for heavy menstrual bleeding. *Eur J Med Res* 2013;18(1):17.
94. Hornyak M, Scholz H, Kohnen R, Bengel J, Kassubek J, Trenkwalder C. What treatment works best for restless legs syndrome? Meta-analyses of dopaminergic and non-dopaminergic medications. *Sleep Med Rev* 2014;18(2):153-64.
95. Howell N, Senanayake E, Freemantle N, Pagano D. Putting the record straight on aprotinin as safe and effective: results from a mixed treatment meta-analysis of trials of aprotinin. *J Thorac Cardiovasc Surg* 2013;145(1):234-40.
96. Karabis A, Lindner L, Mocarski M, Huisman E, Greening A. Comparative efficacy of aclidinium versus glycopyrronium and tiotropium, as maintenance treatment of moderate to severe COPD patients: a systematic review and network meta-analysis*. Int J Chron Obstruct Pulmon Dis* 2013;8:405-23.
97. Landoni G, Greco T, Biondi-Zoccai G, Nigro Neto C, Febres D, Pintaudi M, Pasin L, Cabrini L, Finco G, Zangrillo A. Anaesthetic drugs and survival: a Bayesian network meta-analysis of randomized trials in cardiac surgery. *Br J Anaesth* 2013;111(6):886-96.
98. Leucht S, Cipriani A, Spineli L, Mavridis D, Orey D, Richter F, Samara M, Barbui C, Engel RR, Geddes JR, Kissling W, Stapf MP, Lässig B, Salanti G, Davis JM. Comparative efficacy and tolerability of 15 antipsychotic drugs in schizophrenia: a multiple-treatments meta-analysis. *Lancet* 2013;382(9896):951-62.
99. Levi Marpillat N, Macquin-Mavier I, Tropeano AI, Bachoud-Levi AC, Maison P. Antihypertensive classes, cognitive decline and incidence of dementia: a network meta-analysis. *J Hypertens* 2013;31(6):1073-82.
100. Liao WC, Chien KL, Lin YL, Wu MS, Lin JT, Wang HP, Tu YK. Adjuvant treatments for resected pancreatic adenocarcinoma: a systematic review and network meta-analysis. *Lancet Oncol* 2013;14(11):1095-1103.
101. Malloy RJ, Kanaan AO, Silva MA, Donovan JL. Evaluation of antiplatelet agents for secondary prevention of stroke using mixed treatment comparison meta-analysis. *Clin Ther* 2013;35(10):1490-1500.e7.
102. Migliore A, Broccoli S, Massafra U, Cassol M, Frediani B. Ranking antireabsorptive agents to prevent vertebral fractures in postmenopausal osteoporosis by mixed treatment comparison meta-analysis. *Eur Rev Med Pharmacol Sci* 2013;17(5):658-67.
103. Naci H, Brugts JJ, Fleurence R, Ades AE. Comparative effects of statins on major cerebrovascular events: a multiple-treatments meta-analysis of placebo-controlled and active-comparator trials. *QJM* 2013;106(4):299-306.
104. Navarese EP, Buffon A, Andreotti F, Kozinski M, Welton N, Fabiszak T, Caputo S, Grzesk G, Kubica A, Swiatkiewicz I, Sukiennik A, Kelm M, De Servi S, Kubica J. Meta-analysis of impact of different types and doses of statins on new-onset diabetes mellitus. *Am J Cardiol* 2013;111(8):1123-30.
105. Palmerini T, Biondi-Zoccai G, Della Riva D, Mariani A, Sabaté M, Smits PC, Kaiser C, D'Ascenzo F, Frati G, Mancone M, Genereux P, Stone GW. Clinical outcomes with bioabsorbable polymer- versus durable polymer-based drug-eluting and bare-metal stents: evidence from a comprehensive network meta-analysis. *J Am Coll Cardiol* 2014;63(4):299-307.
106. Rudroju N, Bansal D, Talakokkula ST, Gudala K, Hota D, Bhansali A, Ghai B. Comparative efficacy and safety of six antidepressants and anticonvulsants in painful diabetic neuropathy: a network meta-analysis. *Pain Physician* 2013;16(6):E705-14.
107. Snedecor SJ, Sudharshan L, Cappelleri JC, Sadosky A, Mehta S, Botteman M. Systematic review and meta-analysis of pharmacological therapies for painful diabetic peripheral neuropathy. *Pain Pract* 2014;14(2):167-84.
108. Thorlund K, Mills EJ, Wu P, Ramos E, Chatterjee A, Druyts E, Goadsby PJ. Comparative efficacy of triptans for the abortive treatment of migraine: a multiple treatment comparison meta-analysis. *Cephalalgia* 2014;34(4):258-67.
109. Uthman OA, van der Windt DA, Jordan JL, Dziedzic KS, Healey EL, Peat GM, Foster NE. Exercise for lower limb osteoarthritis: systematic review incorporating trial sequential analysis and network meta-analysis. *BMJ* 2013;347:f5555.
110. Zeppetella G, Davies A, Eijgelshoven I, Jansen JP. A network meta-analysis of the efficacy of opioid analgesics for the management of breakthrough cancer pain episodes. *J Pain Symptom Manage* 2014;47(4):772-785.e5.
111. Bangalore S, Toklu B, Amoroso N, Fusaro M, Kumar S, Hannan EL, Faxon DP, Feit F. Bare metal stents, durable polymer drug eluting stents, and biodegradable polymer drug eluting stents for coronary artery disease: mixed treatment comparison meta-analysis. *BMJ* 2013;347:f6625.
112. Bangalore S, Toklu B, Kotwal A, Volodarskiy A, Sharma S, Kirtane AJ, Feit F. Anticoagulant therapy during primary percutaneous coronary intervention for acute myocardial infarction: a meta-analysis of randomized trials in the era of stents and P2Y12 inhibitors. *BMJ* 2014;349:g6419.
113. Messori A, Fadda V, Maratea D, Trippoli S, Marinai C. Anti-reabsorptive agents in women with osteoporosis: determining statistical equivalence according to evidence-based methods. *J Endocrinol Invest* 2014;37(8):769-773.
114. Messori A, Fadda V, Maratea D, Trippoli S, Gatto R, De Rosa M, Marinai C. Biological drugs for the treatment of moderate-to-severe psoriasis by subcutaneous route: determining statistical equivalence according to evidence-based methods. *Clin Drug Investig* 2014;34(8):593-8.
115. Mills EJ, Thorlund K, Eapen S, Wu P, Prochaska JJ. Cardiovascular events associated with smoking cessation pharmacotherapies: a network meta-analysis. *Circulation* 2014;129(1):28-41.
116. Baji P, Péntek M, Szántó S, Géher P, Gulácsi L, Balogh O, Brodszky V. Comparative efficacy and safety of biosimilar infliximab and other biological treatments in ankylosing spondylitis: systematic literature review and meta-analysis. *Eur J Health Econ* 2014;15 Suppl 1(Suppl 1):S45-52.
117. Oba Y, Lone NA. Comparative efficacy of inhaled corticosteroid and long-acting beta agonist combinations in preventing COPD exacerbations: a Bayesian network meta-analysis. *Int J Chron Obstruct Pulmon Dis* 2014;9:469-79.
118. Johnston BC, Kanters S, Bandayrel K, Wu P, Naji F, Siemieniuk RA, Ball GD, Busse JW, Thorlund K, Guyatt G, Jansen JP, Mills EJ. Comparison of weight loss among named diet programs in overweight and obese adults: a meta-analysis. *JAMA* 2014;312(9):923-33.
119. Dai X, Wang H, Jing Z, Fu P. The effect of a dual combination of noninsulin antidiabetic drugs on lipids: a systematic review and network meta-analysis. *Curr Med Res Opin* 2014;30(9):1777-86.
120. Graudal N, Hubeck-Graudal T, Tarp S, Christensen R, Jürgens G. Effect of combination therapy on joint destruction in rheumatoid arthritis: a network meta-analysis of randomized controlled trials. *PLoS One* 2014;9(9):e106408.
121. Sun F, Wu S, Guo S, Yu K, Yang Z, Li L, Zhang Y, Ji L, Zhan S. Effect of GLP-1 receptor agonists on waist circumference among type 2 diabetes patients: a systematic review and network meta-analysis. *Endocrine* 2015;48(3):794-803.
122. Loveman E, Copley VR, Colquitt JL, Scott DA, Clegg AJ, Jones J, O'Reilly KM, Singh S, Bausewein C, Wells A. The effectiveness and cost-effectiveness of treatments for idiopathic pulmonary fibrosis: systematic review, network meta-analysis and health economic evaluation. *BMC Pharmacol Toxicol* 2014;15:63.
123. Wang H, Yuan J, Hu X, Tao K, Liu J, Hu D. The effectiveness and safety of avanafil for erectile dysfunction: a systematic review and meta-analysis. *Curr Med Res Opin* 2014;30(8):1565-71.
124. Schmid MK, Bachmann LM, Fäs L, Kessels AG, Job OM, Thiel MA. Efficacy and adverse events of aflibercept, ranibizumab and bevacizumab in age-related macular degeneration: a trade-off analysis. *Br J Ophthalmol* 2015;99(2):141-6.
125. Gupta AK, Charrette A. The efficacy and safety of 5α-reductase inhibitors in androgenetic alopecia: a network meta-analysis and benefit-risk assessment of finasteride and dutasteride. *J Dermatolog Treat* 2014;25(2):156-61.
126. Hutchinson M, Fox RJ, Havrdova E, Kurukulasuriya NC, Sarda SP, Agarwal S, Siddiqui MK, Taneja A, Deniz B. Efficacy and safety of BG-12 (dimethyl fumarate) and other disease-modifying therapies for the treatment of relapsing-remitting multiple sclerosis: a systematic review and mixed treatment comparison. *Curr Med Res Opin* 2014;30(4):613-27.
127. Baji P, Péntek M, Czirják L, Szekanecz Z, Nagy G, Gulácsi L, Brodszky V. Efficacy and safety of infliximab-biosimilar compared to other biological drugs in rheumatoid arthritis: a mixed treatment comparison. *Eur J Health Econ* 2014;15 Suppl 1(Suppl 1):S53-64.
128. Zhu Z, Zhang J, Liu Y, Chen M, Guo P, Li K. Efficacy and toxicity of external-beam radiation therapy for localised prostate cancer: a network meta-analysis. *Br J Cancer* 2014;110(10):2396-404.
129. Rollins BM, Silva MA, Donovan JL, Kanaan AO. Evaluation of oral anticoagulants for the extended treatment of venous thromboembolism using a mixed-treatment comparison, meta-analytic approach. *Clin Ther* 2014;36(10):1454-64.e3.
130. Kang N, Sobieraj DM. Indirect treatment comparison of new oral anticoagulants for the treatment of acute venous thromboembolism. *Thromb Res* 2014;133(6):1145-51.
131. Naci H, Dias S, Ades AE. Industry sponsorship bias in research findings: a network meta-analysis of LDL cholesterol reduction in randomised trials of statins. *BMJ* 2014;349:g5741.
132. Gerger H, Munder T, Gemperli A, Nüesch E, Trelle S, Jüni P, Barth J. Integrating fragmented evidence by network meta-analysis: relative effectiveness of psychological interventions for adults with post-traumatic stress disorder. *Psychol Med* 2014;44(15):3151-64.
133. Oba Y, Lone NA. Mortality benefit of vasopressor and inotropic agents in septic shock: a Bayesian network meta-analysis of randomized controlled trials. *J Crit Care* 2014;29(5):706-10.
134. Biondi-Zoccai G, Lotrionte M, Thomsen HS, Romagnoli E, D'Ascenzo F, Giordano A, Frati G. Nephropathy after administration of iso-osmolar and low-osmolar contrast media: evidence from a network meta-analysis. *Int J Cardiol* 2014;172(2):375-80.
135. Vegter S, Tolley K. A network meta-analysis of the relative efficacy of treatments for actinic keratosis of the face or scalp in Europe. *PLoS One* 2014;9(6):e96829.
136. Gupta AK, Daigle D, Lyons DC. Network Meta-analysis of Treatments for Chronic Plaque Psoriasis in Canada. *J Cutan Med Surg* 2014;18(6):371-8.
137. Thorlund K, Wu P, Druyts E, Eapen S, Mills EJ. Nonergot dopamine-receptor agonists for treating Parkinson's disease - a network meta-analysis. *Neuropsychiatr Dis Treat* 2014;10:767-76.
138. Bangalore S, Toklu B, Feit F. Outcomes with coronary artery bypass graft surgery versus percutaneous coronary intervention for patients with diabetes mellitus: can newer generation drug-eluting stents bridge the gap? *Circ Cardiovasc Interv* 2014;7(4):518-25.
139. Griebeler ML, Morey-Vargas OL, Brito JP, Tsapas A, Wang Z, Carranza Leon BG, Phung OJ, Montori VM, Murad MH. Pharmacologic interventions for painful diabetic neuropathy: An umbrella systematic review and comparative effectiveness network meta-analysis. *Ann Intern Med* 2014;161(9):639-49.
140. Wertli MM, Kessels AG, Perez RS, Bachmann LM, Brunner F. Rational pain management in complex regional pain syndrome 1 (CRPS 1)--a network meta-analysis. *Pain Med* 2014;15(9):1575-89.
141. Cucherat M, Stalmans I, Rouland JF. Relative efficacy and safety of preservative-free latanoprost (T2345) for the treatment of open-angle glaucoma and ocular hypertension: an adjusted Indirect comparison meta-analysis of randomized clinical trials. *J Glaucoma* 2014;23(1):e69-75.
142. González-Vacarezza N, Alemán A, González G, Pérez A. Rituximab and tocilizumab for the treatment of rheumatoid arthritis. *Int J Technol Assess Health Care* 2014;30(3):282-8.
143. Cameron C, Coyle D, Richter T, Kelly S, Gauthier K, Steiner S, Carrier M, Coyle K, Bai A, Moulton K, Clifford T, Wells G. Systematic review and network meta-analysis comparing antithrombotic agents for the prevention of stroke and major bleeding in patients with atrial fibrillation. *BMJ Open* 2014;4(6):e004301.
144. Caruba T, Katsahian S, Schramm C, Charles Nelson A, Durieux P, Bégué D, Juillière Y, Dubourg O, Danchin N, Sabatier B. Treatment for stable coronary artery disease: a network meta-analysis of cost-effectiveness studies. *PLoS One* 2014;9(6):e98371.
145. Stagg HR, Zenner D, Harris RJ, Muñoz L, Lipman MC, Abubakar I. Treatment of latent tuberculosis infection: a network meta-analysis. *Ann Intern Med* 2014;161(6):419-28.
146. Wang JC, Tian JH, Ge L, Gan YH, Yang KH. Which is the best Chinese herb injection based on the FOLFOX regimen for gastric cancer? A network meta-analysis of randomized controlled trials. *Asian Pac J Cancer Prev* 2014;15(12):4795-800.
147. Kotb A, Cameron C, Hsieh S, Wells G. Comparative effectiveness of different forms of telemedicine for individuals with heart failure (HF): a systematic review and network meta-analysis. *PLoS One* 2015;10(2):e0118681.
148. Wang X, Wang X, Li S, Meng Z, Liu T, Zhang X. Comparative effectiveness of oral drug therapies for lower urinary tract symptoms due to benign prostatic hyperplasia: a systematic review and network meta-analysis. *PLoS One* 2014;9(9):e107593.
149. Bannuru RR, Schmid CH, Kent DM, Vaysbrot EE, Wong JB, McAlindon TE. Comparative effectiveness of pharmacologic interventions for knee osteoarthritis: a systematic review and network meta-analysis. *Ann Intern Med* 2015;162(1):46-54.
150. Sobieraj DM, Coleman CI, Pasupuleti V, Deshpande A, Kaw R, Hernandez AV. Comparative efficacy and safety of anticoagulants and aspirin for extended treatment of venous thromboembolism: A network meta-analysis. *Thromb Res* 2015;135(5):888-96.
151. Oba Y, Lone NA. Comparative efficacy of long-acting muscarinic antagonists in preventing COPD exacerbations: a network meta-analysis and meta-regression. *Ther Adv Respir Dis* 2015;9(1):3-15.
152. Sun F, Wu S, Wang J, Guo S, Chai S, Yang Z, Li L, Zhang Y, Ji L, Zhan S. Effect of glucagon-like peptide-1 receptor agonists on lipid profiles among type 2 diabetes: a systematic review and network meta-analysis. *Clin Ther* 2015;37(1):225-241.e8.
153. Sun F, Chai S, Li L, Yu K, Yang Z, Wu S, Zhang Y, Ji L, Zhan S. Effects of glucagon-like peptide-1 receptor agonists on weight loss in patients with type 2 diabetes: a systematic review and network meta-analysis. *J Diabetes Res* 2015;2015:157201.
154. Zeng C, Li H, Yang T, Deng ZH, Yang Y, Zhang Y, Lei GH. Electrical stimulation for pain relief in knee osteoarthritis: systematic review and network meta-analysis. *Osteoarthritis Cartilage* 2015;23(2):189-202.
155. Thakur D, Dickerson S, Kumar Bhutani M, Junor R. Impact of prolonged-release oxycodone/naloxone on outcomes affecting patients' daily functioning in comparison with extended-release tapentadol: a systematic review. *Clin Ther* 2015;37(1):212-24.
156. Wang C, Guo L, Chi C, Wang X, Guo L, Wang W, Zhao N, Wang Y, Zhang Z, Li E. Mechanical ventilation modes for respiratory distress syndrome in infants: a systematic review and network meta-analysis. *Crit Care* 2015;19(1):108.
157. Hüttner FJ, Tenckhoff S, Jensen K, Uhlmann L, Kulu Y, Büchler MW, Diener MK, Ulrich A. Meta-analysis of reconstruction techniques after low anterior resection for rectal cancer. *Br J Surg* 2015;102(7):735-45.
158. Xiao J, Wu WX, Ye YY, Lin WJ, Wang L. A Network Meta-analysis of Randomized Controlled Trials Focusing on Different Allergic Rhinitis Medications. *Am J Ther* 2016;23(6):e1568-e1578.
159. Zhu GQ, Shi KQ, Huang S, Huang GQ, Lin YQ, Zhou ZR, Braddock M, Chen YP, Zheng MH. Network meta-analysis of randomized controlled trials: efficacy and safety of UDCA-based therapies in primary biliary cirrhosis. *Medicine (Baltimore)* 2015;94(11):e609.
160. Yildiz A, Nikodem M, Vieta E, Correll CU, Baldessarini RJ. A network meta-analysis on comparative efficacy and all-cause discontinuation of antimanic treatments in acute bipolar mania. *Psychol Med* 2015;45(2):299-317.
161. Stynes G, Svedsater H, Wex J, Lettis S, Leather D, Castelnuovo E, Detry M, Berry S. Once-daily fluticasone furoate/vilanterol 100/25 mcg versus twice daily combination therapies in COPD - mixed treatment comparisons of clinical efficacy. *Respir Res* 2015;16(1):25.
162. Chen L, Staubli SE, Schneider MP, Kessels AG, Ivic S, Bachmann LM, Kessler TM. Phosphodiesterase 5 inhibitors for the treatment of erectile dysfunction: a trade-off network meta-analysis. *Eur Urol* 2015;68(4):674-80.
163. Stevens JW, Khunti K, Harvey R, Johnson M, Preston L, Woods HB, Davies M, Goyder E. Preventing the progression to type 2 diabetes mellitus in adults at high risk: a systematic review and network meta-analysis of lifestyle, pharmacological and surgical interventions. *Diabetes Res Clin Pract* 2015;107(3):320-31.
164. Benedetto U, Raja SG, Albanese A, Amrani M, Biondi-Zoccai G, Frati G. Searching for the second best graft for coronary artery bypass surgery: a network meta-analysis of randomized controlled trials. *Eur J Cardiothorac Surg* 2015;47(1):59-65; discussion 65.
165. Oh GH, Yu JC, Choi KS, Joo EJ, Jeong SH. Simultaneous Comparison of Efficacy and Tolerability of Second-Generation Antipsychotics in Schizophrenia: Mixed-Treatment Comparison Analysis Based on Head-to-Head Trial Data. *Psychiatry Investig* 2015;12(1):46-54.
166. Wang Z, Qiao D, Lu Y, Curtis D, Wen X, Yao Y, Zhao H. Systematic literature review and network meta-analysis comparing bone-targeted agents for the prevention of skeletal-related events in cancer patients with bone metastasis. *Oncologist* 2015;20(4):440-9.
167. Zhu GQ, Shi KQ, Huang S, Wang LR, Lin YQ, Huang GQ, Chen YP, Braddock M, Zheng MH. Systematic review with network meta-analysis: the comparative effectiveness and safety of interventions in patients with overt hepatic encephalopathy. *Aliment Pharmacol Ther* 2015;41(7):624-35.

*Extractable characteristics were less than four*

1. Psaty BM, Smith NL, Siscovick DS, Koepsell TD, Weiss NS, Heckbert SR, Lemaitre RN, Wagner EH, Furberg CD. Health outcomes associated with antihypertensive therapies used as first-line agents. A systematic review and meta-analysis. *JAMA* 1997;277(9):739-45.
2. Yu CH, Beattie WS. The effects of volatile anesthetics on cardiac ischemic complications and mortality in CABG: a meta-analysis. *Can J Anaesth* 2006;53(9):906-18.
3. Singh JA, Christensen R, Wells GA, Suarez-Almazor ME, Buchbinder R, Lopez-Olivo MA, Ghogomu ET, Tugwell P. A network meta-analysis of randomized controlled trials of biologics for rheumatoid arthritis: a Cochrane overview. *CMAJ* 2009;181(11):787-96.
4. Roskell NS, Lip GY, Noack H, Clemens A, Plumb JM. Treatments for stroke prevention in atrial fibrillation: a network meta-analysis and indirect comparisons versus dabigatran etexilate. *Thromb Haemost* 2010;104(6):1106-15.
5. Sciarretta S, Palano F, Tocci G, Baldini R, Volpe M. Antihypertensive treatment and development of heart failure in hypertension: a Bayesian network meta-analysis of studies in patients with hypertension and high cardiovascular risk. *Arch Intern Med* 2011;171(5):384-94.
6. Stowe R, Ives N, Clarke CE, Handley K, Furmston A, Deane K, van Hilten JJ, Wheatley K, Gray R. Meta-analysis of the comparative efficacy and safety of adjuvant treatment to levodopa in later Parkinson's disease. *Mov Disord* 2011;26(4):587-98.
7. Coleman CI, Baker WL, Kluger J, White CM. Antihypertensive medication and their impact on cancer incidence: a mixed treatment comparison meta-analysis of randomized controlled trials. *J Hypertens* 2008;26(4):622-9.
8. Eisenberg MJ, Filion KB, Yavin D, Bélisle P, Mottillo S, Joseph L, Gervais A, O'Loughlin J, Paradis G, Rinfret S, Pilote L. Pharmacotherapies for smoking cessation: a meta-analysis of randomized controlled trials. *CMAJ* 2008;179(2):135-44.
9. Peterson K, McDonagh MS, Fu R. Comparative benefits and harms of competing medications for adults with attention-deficit hyperactivity disorder: a systematic review and indirect comparison meta-analysis. *Psychopharmacology (Berl)* 2008;197(1):1-11.
10. Hofmeyr GJ, Gülmezoglu AM, Novikova N, Linder V, Ferreira S, Piaggio G. Misoprostol to prevent and treat postpartum haemorrhage: a systematic review and meta-analysis of maternal deaths and dose-related effects. *Bull World Health Organ* 2009;87(9):666-77.
11. Lapitan MC, Cody JD, Grant A. Open retropubic colposuspension for urinary incontinence in women. *Cochrane Database Syst Rev* 2009;(4):CD002912. doi: 10.1002/14651858.CD002912.pub4.
12. Mills EJ, Perri D, Cooper C, Nachega JB, Wu P, Tleyjeh I, Phillips P. Antifungal treatment for invasive Candida infections: a mixed treatment comparison meta-analysis. *Ann Clin Microbiol Antimicrob* 2009;8:23.
13. Mills EJ, Rachlis B, O'Regan C, Thabane L, Perri D. Metastatic renal cell cancer treatments: an indirect comparison meta-analysis. *BMC Cancer* 2009;9:34.
14. Meader N. A comparison of methadone, buprenorphine and alpha(2) adrenergic agonists for opioid detoxification: a mixed treatment comparison meta-analysis. *Drug Alcohol Depend* 2010;108(1-2):110-4.
15. Uthman OA, Abdulmalik J. Comparative efficacy and acceptability of pharmacotherapeutic agents for anxiety disorders in children and adolescents: a mixed treatment comparison meta-analysis. *Curr Med Res Opin* 2010;26(1):53-9.
16. Blanchard P, Hill C, Guihenneuc-Jouyaux C, Baey C, Bourhis J, Pignon JP; MACH-NC and MARCH Collaborative Groups. Mixed treatment comparison meta-analysis of altered fractionated radiotherapy and chemotherapy in head and neck cancer. *J Clin Epidemiol* 2011;64(9):985-92.
17. Thakkinstian A, Attia J, Anothaisintawee T, Nickel JC. α-blockers, antibiotics and anti-inflammatories have a role in the management of chronic prostatitis/chronic pelvic pain syndrome. *BJU Int* 2012;110(7):1014-22.
18. Zagmutt FJ, Tarrants ML. Indirect comparisons of adverse events and dropout rates in early Parkinson's disease trials of pramipexole, ropinirole, and rasagiline. *Int J Neurosci* 2012;122(7):345-53.
19. Desai RJ, Hansen RA, Rao JK, Wilkins TM, Harden EA, Yuen A, Jonas DE, Roubey R, Jonas B, Gartlehner G, Lux L, Donahue KE. Mixed treatment comparison of the treatment discontinuations of biologic disease-modifying antirheumatic drugs in adults with rheumatoid arthritis. *Ann Pharmacother* 2012;46(11):1491-505.
20. Chang KV, Chen SY, Chen WS, Tu YK, Chien KL. Comparative effectiveness of focused shock wave therapy of different intensity levels and radial shock wave therapy for treating plantar fasciitis: a systematic review and network meta-analysis. *Arch Phys Med Rehabil* 2012;93(7):1259-68.
21. Fretheim A, Odgaard-Jensen J, Brørs O, Madsen S, Njølstad I, Norheim OF, Svilaas A, Kristiansen IS, Thürmer H, Flottorp S. Comparative effectiveness of antihypertensive medication for primary prevention of cardiovascular disease: systematic review and multiple treatments meta-analysis. *BMC Med* 2012;10:33.
22. Hutton B, Joseph L, Fergusson D, Mazer CD, Shapiro S, Tinmouth A. Risks of harms using antifibrinolytics in cardiac surgery: systematic review and network meta-analysis of randomised and observational studies. *BMJ* 2012;345:e5798.
23. Ramsberg J, Asseburg C, Henriksson M. Effectiveness and cost-effectiveness of antidepressants in primary care: a multiple treatment comparison meta-analysis and cost-effectiveness model. *PLoS One* 2012;7(8):e42003.
24. Wu MS, Tan SC, Xiong T. Indirect comparison of randomised controlled trials: comparative efficacy of dexlansoprazole vs. esomeprazole in the treatment of gastro-oesophageal reflux disease. *Aliment Pharmacol Ther* 2013;38(2):190-201.
25. Messori A, Fadda V, Maratea D, Trippoli S, Gatto R, De Rosa M, Marinai C. Biological drugs for the treatment of rheumatoid arthritis by the subcutaneous route: interpreting efficacy data to assess statistical equivalence. *Ther Adv Musculoskelet Dis* 2014;6(6):207-16.
26. Zhang YW, Zhang YL, Pan H, Wei FX, Zhang YC, Shao Y, Han W, Liu HP, Wang ZY, Yang SH. Chemotherapy for patients with gastric cancer after complete resection: a network meta-analysis. *World J Gastroenterol* 2014;20(2):584-92.
27. Gresham GK, Wells GA, Gill S, Cameron C, Jonker DJ. Chemotherapy regimens for advanced pancreatic cancer: a systematic review and network meta-analysis. *BMC Cancer* 2014;14:471.
28. Dai N, Xu D, Zhang J, Wei Y, Li W, Fan B, Xu Y. Different β-blockers and initiation time in patients undergoing noncardiac surgery: a meta-analysis. *Am J Med Sci* 2014;347(3):235-44.
29. Dal Molin A, Allara E, Montani D, Milani S, Frassati C, Cossu S, Tonella S, Brioschi D, Rasero L. Flushing the central venous catheter: is heparin necessary? *J Vasc Access* 2014;15(4):241-8.
30. Liang W, Wu X, Fang W, Zhao Y, Yang Y, Hu Z, Xue C, Zhang J, Zhang J, Ma Y, Zhou T, Yan Y, Hou X, Qin T, Dinglin X, Tian Y, Huang P, Huang Y, Zhao H, Zhang L. Network meta-analysis of erlotinib, gefitinib, afatinib and icotinib in patients with advanced non-small-cell lung cancer harboring EGFR mutations. *PLoS One* 2014;9(2):e85245.
31. Chambers JD, Winn A, Zhong Y, Olchanski N, Cangelosi MJ. Potential role of network meta-analysis in value-based insurance design. *Am J Manag Care* 2014;20(8):641-8.
32. Stidham RW, Lee TC, Higgins PD, Deshpande AR, Sussman DA, Singal AG, Elmunzer BJ, Saini SD, Vijan S, Waljee AK. Systematic review with network meta-analysis: the efficacy of anti-tumour necrosis factor-alpha agents for the treatment of ulcerative colitis. *Aliment Pharmacol Ther* 2014;39(7):660-71.
33. Ashby RL, Gabe R, Ali S, Saramago P, Chuang LH, Adderley U, Bland JM, Cullum NA, Dumville JC, Iglesias CP, Kang'ombe AR, Soares MO, Stubbs NC, Torgerson DJ. VenUS IV (Venous leg Ulcer Study IV) - compression hosiery compared with compression bandaging in the treatment of venous leg ulcers: a randomised controlled trial, mixed-treatment comparison and decision-analytic model. *Health Technol Assess* 2014;18(57):1-293, v-vi.

*No information on the studies’ clinical and methodological characteristics*

1. Elliott WJ, Meyer PM. Incident diabetes in clinical trials of antihypertensive drugs: a network meta-analysis. *Lancet* 2007;369(9557):201-7.
2. Thijs V, Lemmens R, Fieuws S. Network meta-analysis: simultaneous meta-analysis of common antiplatelet regimens after transient ischaemic attack or stroke. *Eur Heart J* 2008;29(9):1086-92.
3. Sultana A, Ghaneh P, Cunningham D, Starling N, Neoptolemos JP, Smith CT. Gemcitabine based combination chemotherapy in advanced pancreatic cancer-indirect comparison. *BMC Cancer* 2008;8:192.
4. van der Valk R, Webers CA, Lumley T, Hendrikse F, Prins MH, Schouten JS. A network meta-analysis combined direct and indirect comparisons between glaucoma drugs to rank effectiveness in lowering intraocular pressure. *J Clin Epidemiol* 2009;62(12):1279-83.
5. Welton NJ, Caldwell DM, Adamopoulos E, Vedhara K. Mixed treatment comparison meta-analysis of complex interventions: psychological interventions in coronary heart disease. *Am J Epidemiol* 2009;169(9):1158-65.
6. Edwards SJ, Lind T, Lundell L, DAS R. Systematic review: standard- and double-dose proton pump inhibitors for the healing of severe erosive oesophagitis -- a mixed treatment comparison of randomized controlled trials. *Aliment Pharmacol Ther* 2009;30(6):547-56.
7. Cipriani A, Furukawa TA, Salanti G, Geddes JR, Higgins JP, Churchill R, Watanabe N, Nakagawa A, Omori IM, McGuire H, Tansella M, Barbui C. Comparative efficacy and acceptability of 12 new-generation antidepressants: a multiple-treatments meta-analysis. *Lancet* 2009;373(9665):746-58.
8. Edwards SJ, Clarke MJ, Wordsworth S, Welton NJ. Carbapenems versus other beta-lactams in the treatment of hospitalised patients with infection: a mixed treatment comparison. *Curr Med Res* Opin 2009;25(1):251-61.
9. Golfinopoulos V, Pentheroudakis G, Salanti G, Nearchou AD, Ioannidis JP, Pavlidis N. Comparative survival with diverse chemotherapy regimens for cancer of unknown primary site: multiple-treatments meta-analysis. *Cancer Treat Rev* 2009;35(7):570-3.
10. Owen A. Antithrombotic treatment for the primary prevention of stroke in patients with non valvular atrial fibrillation: a reappraisal of the evidence and network meta analysis. *Int J Cardiol* 2010;142(3):218-23.
11. Salliot C, Finckh A, Katchamart W, Lu Y, Sun Y, Bombardier C, Keystone E. Indirect comparisons of the efficacy of biological antirheumatic agents in rheumatoid arthritis in patients with an inadequate response to conventional disease-modifying antirheumatic drugs or to an anti-tumour necrosis factor agent: a meta-analysis. *Ann Rheum Dis* 2011;70(2):266-71.
12. Virgili G, Novielli N, Menchini F, Murro V, Giacomelli G. Pharmacological treatments for neovascular age-related macular degeneration: can mixed treatment comparison meta-analysis be useful? *Curr Drug Targets* 2011;12(2):212-20.
13. Costa J, Fareleira F, Ascenção R, Borges M, Sampaio C, Vaz-Carneiro A. Clinical comparability of the new antiepileptic drugs in refractory partial epilepsy: a systematic review and meta-analysis. *Epilepsia* 2011;52(7):1280-91.
14. Maratea D, Fadda V, Trippoli S, Messori A. Prevention of venous thromboembolism after major orthopedic surgery: indirect comparison of three new oral anticoagulants. *J Thromb Haemost* 2011;9(9):1868-70.
15. Gartlehner G, Hansen RA, Morgan LC, Thaler K, Lux L, Van Noord M, Mager U, Thieda P, Gaynes BN, Wilkins T, Strobelberger M, Lloyd S, Reichenpfader U, Lohr KN. Comparative benefits and harms of second-generation antidepressants for treating major depressive disorder: an updated meta-analysis. *Ann Intern Med* 2011;155(11):772-85.
16. Makani H, Bangalore S, Romero J, Wever-Pinzon O, Messerli FH. Effect of renin-angiotensin system blockade on calcium channel blocker-associated peripheral edema. *Am J Med* 2011;124(2):128-35.
17. Maund E, McDaid C, Rice S, Wright K, Jenkins B, Woolacott N. Paracetamol and selective and non-selective non-steroidal anti-inflammatory drugs for the reduction in morphine-related side-effects after major surgery: a systematic review. *Br J Anaesth* 2011;106(3):292-7.
18. Del Santo F, Maratea D, Fadda V, Trippoli S, Messori A. Treatments for relapsing-remitting multiple sclerosis: summarising current information by network meta-analysis. *Eur J Clin Pharmacol* 2012;68(4):441-8.
19. Trinquart L, Abbé A, Ravaud P. Impact of reporting bias in network meta-analysis of antidepressant placebo-controlled trials. *PLoS One* 2012;7(4):e35219.
20. Mavranezouli I, Meader N, Cape J, Kendall T. The cost effectiveness of pharmacological treatments for generalized anxiety disorder. *Pharmacoeconomics* 2013;31(4):317-33.
21. Kwak HJ, Kim JY, Kim YB, Min SK, Moon BK, Kim JY. Pharmacological prevention of rocuronium-induced injection pain or withdrawal movements: a meta-analysis. *J Anesth* 2013;27(5):742-9.
22. Tadrous M, Mamdani MM, Juurlink DN, Krahn MD, Lévesque LE, Cadarette SM. Comparative gastrointestinal safety of bisphosphonates in primary osteoporosis: a network meta-analysis-reply to Pazianas and Abrahamsen. *Osteoporos Int* 2014;25(11):2671-2.
23. Dong J, Gao L, Lu W, Xu Z, Zheng J. Pharmacological interventions for acceleration of the onset time of rocuronium: a meta-analysis. *PLoS One* 2014;9(12):e114231.
24. Signorovitch JE, Betts KA, Yan YS, LeReun C, Sundaram M, Wu EQ, Mulani P. Comparative efficacy of biological treatments for moderate-to-severe psoriasis: a network meta-analysis adjusting for cross-trial differences in reference arm response. *Br J Dermatol* 2015;172(2):504-12.
25. Wang L, Baser O, Kutikova L, Page JH, Barron R. The impact of primary prophylaxis with granulocyte colony-stimulating factors on febrile neutropenia during chemotherapy: a systematic review and meta-analysis of randomized controlled trials. *Support Care Cancer* 2015;23(11):3131-40.

*Characteristics were summarised per treatment or across studies*

1. Picard P, Tramèr MR. Prevention of pain on injection with propofol: a quantitative systematic review. *Anesth Analg* 2000;90(4):963-9.
2. Singh JA, Wells GA, Christensen R, Tanjong Ghogomu E, Maxwell L, Macdonald JK, Filippini G, Skoetz N, Francis D, Lopes LC, Guyatt GH, Schmitt J, La Mantia L, Weberschock T, Roos JF, Siebert H, Hershan S, Lunn MP, Tugwell P, Buchbinder R. Adverse effects of biologics: a network meta-analysis and Cochrane overview. *Cochrane Database Syst Rev* 2011;2011(2):CD008794.
3. Hartling L, Fernandes RM, Bialy L, Milne A, Johnson D, Plint A, Klassen TP, Vandermeer B. Steroids and bronchodilators for acute bronchiolitis in the first two years of life: systematic review and meta-analysis. *BMJ* 2011;342:d1714.
4. Rheims S, Perucca E, Cucherat M, Ryvlin P. Factors determining response to antiepileptic drugs in randomized controlled trials. A systematic review and meta-analysis. *Epilepsia* 2011;52(2):219-33.
5. Shamliyan TA, Choi JY, Ramakrishnan R, Miller JB, Wang SY, Taylor FR, Kane RL. Preventive pharmacologic treatments for episodic migraine in adults. J Gen Intern Med 2013;28(9):1225-37.

*Article in Hungarian*

1. Brodszky V. A rheumatoid arthritisben alkalmazott biológiai kezelések hatásossága az ACR70-végpont szerint: indirekt összehasonlítás és metaregresszió Bayes-modell alkalmazásával [Effectiveness of biological treatments based on ACR70 response in rheumatoid arthritis: indirect comparison and meta-regression using Bayes-model]. *Orv Hetil* 2011;152(23):919-28.

*Article in Chinese*

1. Zhang He-na, Shao Rong. A Network Meta-Analysis on the Influence of Antihypertensive Agents on New-onset Diabetes. *Chin Pharm J* 2013;48(11):930-933. <https://doi.org/10.11669/cpj.2013.11.021>

*Characteristics were reported in textual format*

1. Daniels JP, Middleton LJ, Champaneria R, Khan KS, Cooper K, Mol BW, Bhattacharya S; International Heavy Menstrual Bleeding IPD Meta-analysis Collaborative Group. Second generation endometrial ablation techniques for heavy menstrual bleeding: network meta-analysis. *BMJ* 2012;344:e2564.

*Network connectivity was lost after removing studies with missing data*

1. Donahue KE, Jonas DE, Hansen RA, Roubey R, Jonas B, Lux LJ, Gartlehner G, Harden E, Wilkins T, Peravali V, Bangdiwala SI, Yuen A, Thieda P, Morgan LC, Crotty K, Desai R, Van Noord M. Drug Therapy for Rheumatoid Arthritis in Adults: An Update [Internet]. Rockville (MD): Agency for Healthcare Research and Quality (US); 2012 Apr. Report No.: 12-EHC025-EF.

*No study names or references were provided by nmadb*

1. Murad MH, Drake MT, Mullan RJ, Mauck KF, Stuart LM, Lane MA, Abu Elnour NO, Erwin PJ, Hazem A, Puhan MA, Li T, Montori VM. Clinical review. Comparative effectiveness of drug treatments to prevent fragility fractures: a systematic review and network meta-analysis. *J Clin Endocrinol Metab* 2012;97(6):1871-80.

*No response from the corresponding author to share the requested data*

1. Zintzaras E, Miligkos M, Ziakas P, Balk EM, Mademtzoglou D, Doxani C, Mprotsis T, Gowri R, Xanthopoulou P, Mpoulimari I, Kokkali C, Dimoulou G, Rodopolou P, Stefanidis I, Kent DM, Hadjigeorgiou GM. Assessment of the relative effectiveness and tolerability of treatments of type 2 diabetes mellitus: a network meta-analysis. *Clin Ther* 2014;36(10):1443-53.e9.
