# Supplementary material for: A database to initiate methodological advances in the evaluation of transitivity assumption in network meta-analysis: qualitative features and limitations of the tracenma R package
Source: BMC Med Res Methodol. 2025 Jul 31;25:183. doi: 10.1186/s12874-025-02634-x (PMC12315375; doi:10.1186/s12874-025-02634-x)
Supplement: Supplementary file 2 — Additional file 2. Illustrating transitivity evaluation with tracenma [file 12874_2025_2634_MOESM2_ESM.docx]

**Supporting Information for the article 'A database to initiate methodological advances in the evaluation of transitivity assumption in network meta-analysis: qualitative features and limitations of the tracenma R package'**

Loukia M. Spineli^1^  [Spineli.Loukia@mh-hannover.de](mailto:Spineli.Loukia@mh-hannover.de)

Andrés Mauricio García-Sierra^2^ [andresmauriciog@uchicago.edu](mailto:andresmauriciog@uchicago.edu)

Juan Jose Yepes-Nuñez^3,4^  [jj.yepesn@uniandes.edu.co](mailto:jj.yepesn@uniandes.edu.co)

^1^Midwifery Research and Education Unit, Hannover Medical School, Hannover, Germany

^2^Department of Public Health Sciences, University of Chicago, Chicago, USA

^3^School of Medicine, Universidad de los Andes, Bogotá D.C., Colombia

^4^Internal Medicine Department, Fundación Santa Fe de Bogotá, Bogotá D.C., Colombia

**Illustrating transitivity evaluation with tracenma**

We illustrate the synergy of the tracenma R package and currently available methods to assess transitivity: multiple statistical tests, graphical evaluation, and study dissimilarities based on Gower's dissimilarity (GD) coefficient [1], a newly proposed approach for transitivity assessment. The tracenma R package retrieves several networks with extracted study-level aggregate clinical and methodological characteristics. Then, current and new methods for transitivity assessment can be ***evaluated empirically*** using the tracenma R package. Please refer to the official repository of tracenma (<https://loukiaspin.github.io/tracenma/>) to navigate to the functionalities and output of this R package.

1. **Install and load the tracenma R package**

Copy-paste to an R script and run the following code to install and load the tracenma R package:

install.packages("tracenma")

library(tracenma)

1. **Choose a dataset to work on**

We will use the network of Vestergaard et al. [2] with 12 studies on concomitant treatment with calcium and/vitamin D as therapy for bone mineral density. The data have been extracted from Table 1 of the systematic review report [2]. Run the following code to retrieve the corresponding dataset from the tracenma R package:

example0 <- get.dataset(pmid = 16951908)$Dataset

# Turn into data-frame

(example <- as.data.frame(example0))

And this is the output: a dataset of 12 studies with seven aggregate characteristics: sex, sample.size, h.rPTH, calcium, vitamin.D, duration, and quality.

> example

trial treat1 treat2 arm1 arm2 sex sample.size h.rPTH calcium vitamin.D duration quality

1 Finkelstein 1998 (26) 1 2 control PTH 40 female 43 1-34 NA NA 12 2

2 Lane 1998 (42) 1 3 control PTH 25 female 51 1-34 1500 800 12 3

3 Kurland 2000 (33) 1 4 control PTH 32 male 23 1-34 1500 400 18 3

4 Cosman 2001 (24) 1 3 control PTH 25 female 52 1-34 1500 800 36 3

5 Neer 2001 (5) 1 2 control PTH 40 female 882 1-34 1000 400 21 3

6 Neer 2001 (5) 1 5 control PTH 20 female 892 1-34 1000 400 21 3

7 Neer 2001 (5) 2 5 PTH 40 PTH 20 female 878 1-34 1000 400 21 3

8 Body 2002 (43) 1 2 control PTH 40 female 146 1-34 1000 400 12 4

9 Finkelstein 2003 (28) 1 2 control PTH 40 male 48 1-34 1000 400 30 3

10 Finkelstein 2003 (28) 1 6 control PTH+ALN male 53 1-34 1000 400 30 3

11 Finkelstein 2003 (28) 2 6 PTH 40 PTH+ALN male 45 1-34 1000 400 30 3

12 Hodsman 2003 (32) 1 7 control PTH 100 female 104 1-84 500 400 12 4

13 Hodsman 2003 (32) 1 8 control PTH 75 female 105 1-84 500 400 12 4

14 Hodsman 2003 (32) 1 9 control PTH 50 female 103 1-84 500 400 12 4

15 Hodsman 2003 (32) 7 8 PTH 100 PTH 75 female 103 1-84 500 400 12 4

16 Hodsman 2003 (32) 7 9 PTH 100 PTH 50 female 101 1-84 500 400 12 4

17 Hodsman 2003 (32) 8 9 PTH 75 PTH 50 female 102 1-84 500 400 12 4

18 Orwoll 2003 (6) 1 2 control PTH 40 male 286 1-34 1000 400 11 4

19 Orwoll 2003 (6) 1 5 control PTH 20 male 298 1-34 1000 400 11 4

20 Orwoll 2003 (6) 2 5 PTH 40 PTH 20 male 290 1-34 1000 400 11 4

21 Black 2003 (23) 1 6 control PTH+ALN female 119 1-84 500 400 12 4

22 Black 2003 (23) 1 7 control PTH 100 female 179 1-84 500 400 12 4

23 Black 2003 (23) 6 7 PTH+ALN PTH 100 female 178 1-84 500 400 12 4

24 Cosman 2005 (25) 1 3 control PTH 25 female 126 1-34 1200 600 15 3

25 McClung 2005 1 5 control PTH 20 female 203 1-34 1000 400 12 3

The following text describes the structure of the dataset; you may skip it and go directly to the R code below. The first column (trial) refers to the names of the included studies. The second and third columns (treat1 and treat2, respectively) refer to the baseline (or control) and non-baseline (or experimental) treatments as identifying numbers (the numbers in treat1 are consistently smaller than those in treat2). The fourth and fifth columns (arm1 and arm2, respectively) present the names of the corresponding treatments. The remaining seven columns refer to the extracted aggregate characteristics: sex, sample.size, h.rPTH, calcium, vitamin.D, duration, and quality. There are more than 12 rows because there are five multi-arm studies: Neer 2001 (5), Finkelstein 2003 (28), Orwoll 2003 (6), and Black 2003 (23) are three-arm studies, and Hodsman 2003 (32) is a four-arm study, leading to six possible pairwise comparisons (and hence, six rows in the dataset). Therefore, the aggregate characteristics are reported for each possible pairwise comparison of each study. When a study misses a characteristic, NA appears in the corresponding part of the dataset; for instance, calcium and vitamid.D for the study Finkelstein 1998 (26).

1. **Some tiny data manipulation**

Transitivity assessment includes assessing the distribution of important effect modifiers *across* the observed pairwise comparisons. Run the following code to create an extra column with the observed pairwise comparisons and check their frequency:

example$compars <- paste(example$ arm2, "vs", example$ arm1)

table(example$compars)

There are eight comparisons informed by only one study; hence, statistical tests and graphical evaluation of transitivity may not work for this network:

> table(table(example$compars))

1 2 3 5

8 3 2 1

1. **Current available methods for transitivity assessment**

A recent systematic survey of published systematic reviews with network meta-analysis revealed a poor evaluation of the transitivity assumption overall for lacking transparency and reproducibility in the methods employed [3]. Among the systematic reviews claiming to have assessed transitivity, most compared narratively the observed pairwise comparisons regarding the investigated clinical and methodological characteristics [3]. Only a few systematic reviews evaluated transitivity transparently by testing the association between each characteristic and the observed comparisons (for instance, Table 1 in Schwarz et al. [4]) or visualising the distribution of each characteristic across the observed comparisons (for instance, Figures 15 and 16 in Sbidian et al. [5]) [3]. A recent methodological study proposed a novel approach to assess the transitivity assumption based on study dissimilarities for study-level aggregate clinical and methodological characteristics that act as important effect modifiers [1]. Contrary to multiple testing and graphical evaluation of the transitivity assumption, this approach does not rely on hypothesis testing (with implications for multiplicity due to multiple testing) or subjective judgments; instead, it is grounded on a well-established dissimilarity measure, the GD coefficient, offering a semi-objective exploration of the transitivity assumption [1]. In the following sections, we illustrate assessing transitivity using (i) multiple statistical testing, (ii) graphical evaluation, and (iii) the GD metric.

- 1. **Multiple statistical tests**

The dataset comprises two categorical characteristics (sex and h.rPTH), with the remaining characteristics being metric. Hence, we perform Welch's ANOVA for each metric characteristic and the chi-squared test for each categorical characteristic. The characteristic is the dependent variable, and the observed comparisons are the independent variable. The null hypothesis states no association between the characteristic and the observed comparisons. We use the functions chisq.test and oneway.test from the stats R package.

Run the following code to perform the chi-squared test for sex and h.rPTH and get the results:

# Sex

chisq.test(table(example$sex, example$compars))

> chisq.test(table(example$sex, example$compars))

Pearson's Chi-squared test

data: table(example$sex, example$compars)

X-squared = 10.78, df = 13, p-value = 0.6292

Warning:

In chisq.test(table(example$sex, example$compars)) :

Chi-squared approximation may be incorrect

# PTH therapy duration

chisq.test(table(example$h.rPTH, example$compars))

> chisq.test(table(example$h.rPTH, example$compars))

Pearson's Chi-squared test

data: table(example$h.rPTH, example$compars)

X-squared = 22.83, df = 13, p-value = 0.04376

Warning:

In chisq.test(table(example$sex, example$compars)) :

Chi-squared approximation may be incorrect

There is a statistically significant association only for h.rPTH (p-value < 0.05), implying systematic variability of this characteristic across the observed comparisons; however, none of the chi-squared tests was credible for having small expected values in all combinations.

Run the following code to perform Welsh's ANOVA for the remaining (metric) characteristics:

# Sample size

oneway.test(example$sample.size ~ example$compars, var.equal = FALSE)

# Calcium

oneway.test(example$calcium ~ example$compars, var.equal = FALSE)

# Vitamin D

oneway.test(example$vitamin.D ~ example$compars, var.equal = FALSE)

# Study duration (in months)

oneway.test(example$duration ~ example$compars, var.equal = FALSE)

# Study quality using the Jadad scale

oneway.test(example$quality ~ example$compars, var.equal = FALSE)

None of the tests could be performed due to single-study comparisons, as we got the error message 'not enough observations'.

**Interpretation**

Statistical testing could not offer a sufficient transitivity evaluation in the investigated network. In networks with single-study comparisons, statistical tests for the plausibility of transitivity will not be feasible. Furthermore, p-values exceeding a selected significance level may invite misinterpreting statistical non-significant associations as evidence of transitivity. A recent study revealed several challenges with statistical tests to evaluate transitivity [1]. The tracenma R package was used to empirically assess (commonly applied) statistical tests for transitivity and uncover their limitations [1].

- 1. **Graphical evaluation of transitivity**

We will use the function distr_characteristics from the rnmamod R package [6] to create (pretty) violin plots with integrated box plots and dots for the metric characteristics and stacked bar plots for the categorical characteristics.

Initially, we make some necessary transformations to a few columns to apply the function:

# Turn the first three columns (trial and arms) into character

example[, 1:3] <- lapply(example[, 1:3], as.character)

# Turn character into factor for the corresponding characteristics

example[, c(6, 8)] <- lapply(example[, c(6, 8)], as.factor)

Then, run the following code to install and load the rnmamod R package:

install.packages("rnmamod")

library(rnmamod)

Finally, run the following code to obtain the graphs for each characteristic:

distr_characteristics(input = example[, -c(4, 5, 13)],

drug_names = as.character(1:9),

rename_char = list(4:10, c("Sex", "Sample size",

"PTH treatment duration", "Calcium ", "Vitamin D", "Study duration", "Study quality")),

label_size = 3,

title_size = 9.5,

axis_title_size = 9.5,

axis_text_size = 9.5,

axis_x_text_angle = 0,

legend_text_size = 9.5)

# Find out more about this function

?distr_characteristics

The function returns a list of plots for each characteristic, with the x-axis referring to the observed pairwise comparisons and the y-axis referring to the absolute frequency (counts) or range of values for the categorical or metric characteristic, respectively. The distribution of study duration and sex across the observed pairwise comparisons is presented below:


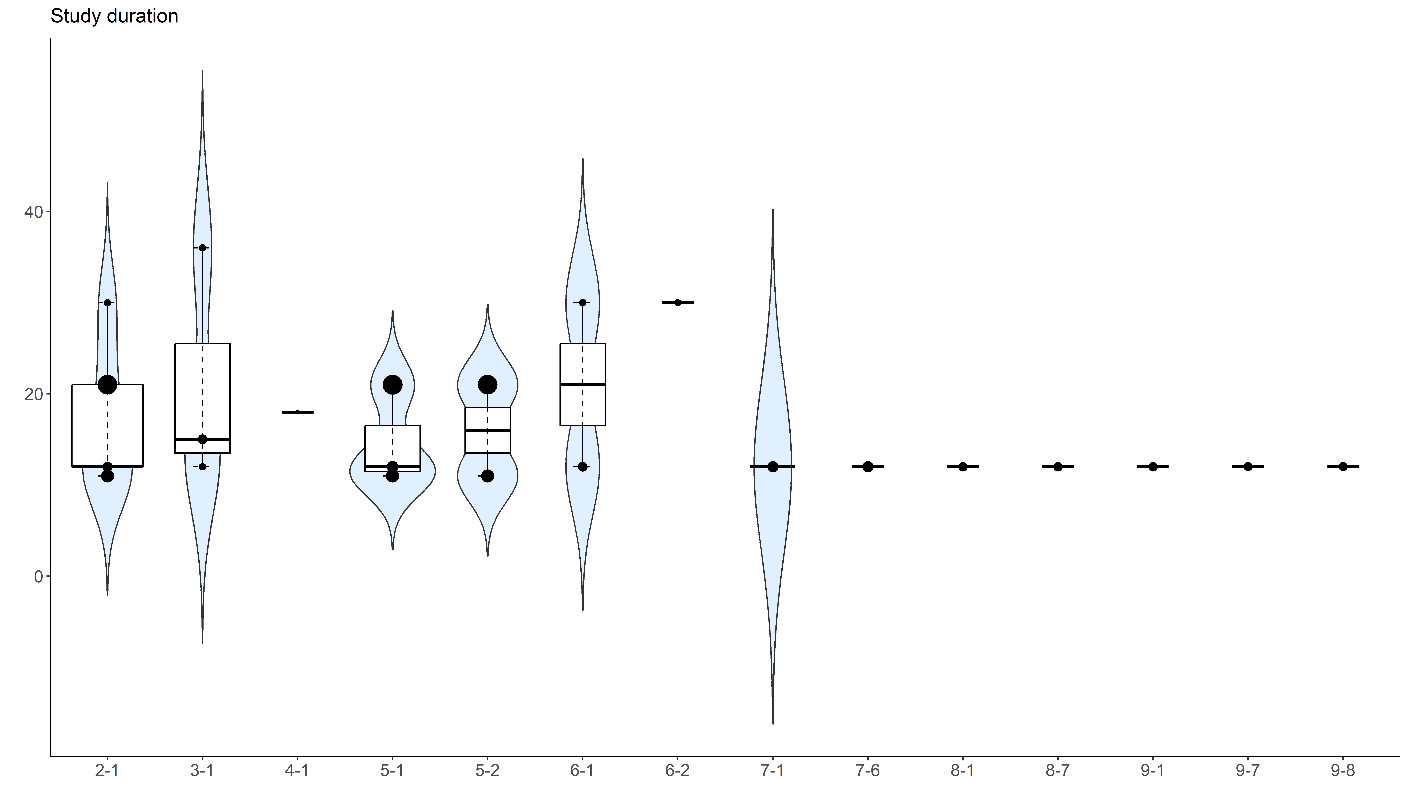


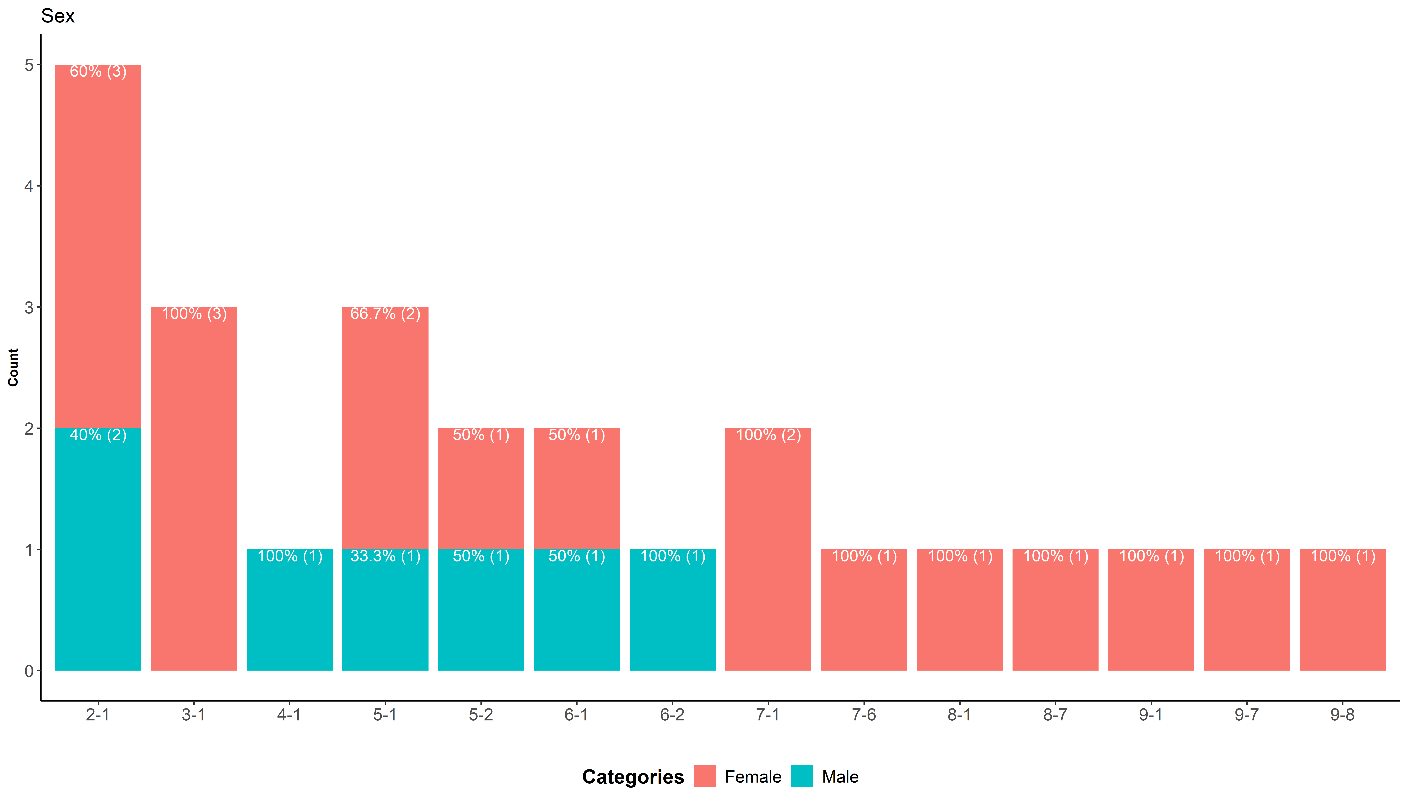


**Interpretation**

Comparisons informed by more than one study covered a great range of study durations: comparisons 5-1 and 5-2 had a similarly narrow range of study durations, and comparisons 2-1, 3-1 and 6-1 covered a relatively wider range of study durations. The single-study comparisons with the same study duration refer to the network's four-arm trial (1 versus 7 versus 8 versus 9). Furthermore, apart from the four-arm trial that included only female participants, the remaining comparisons had female and male participants, with comparisons 2-1 and 5-1 mainly having female participants. It is not straightforward to infer in favour or against transitivity, and the presence of single-study comparisons complicates the decision. In sparse networks, a graphical evaluation of transitivity would not be straightforward, inviting overly subjective conclusions.

- 1. **Gower's dissimilarity metric (recently proposed)**

We use the comp_clustering function from the rnmamod R package [6] to calculate the study dissimilarities based on the seven extracted characteristics, which we assume are important effect modifiers relevant for the transitivity evaluation. The following code uses the default arguments of the comp_clustering function and the threshold for a general health setting [1]:

comp_clustering(input = example[, -c(4, 5, 13)],

threshold = 0.13,

get_plots = TRUE)

# Find out more about this function

?comp_clustering

The function returns a rich output, including results on the console and several plots. Initially, a message printed in red appears on the console regarding the number of observed comparisons and those informed by one study only, as well as any characteristics that were dropped due to many missing data (no characteristics were dropped):

- 14 observed comparisons (8 single-study comparisons)

- Dropped characteristics: none

Then, two symmetric tables appear on the console: one on the study dissimilarities ($Trials_diss_table) and another on the overall dissimilarities for each comparison and pair of comparisons ($Comparisons_diss_table). The first table is also illustrated in two separate figures:

- violin plot with integrated box plots and dots for the *main diagonal elements*, with each element (i.e., observed comparison) being a violin ($Within_comparison_dissimilarity), as shown below:


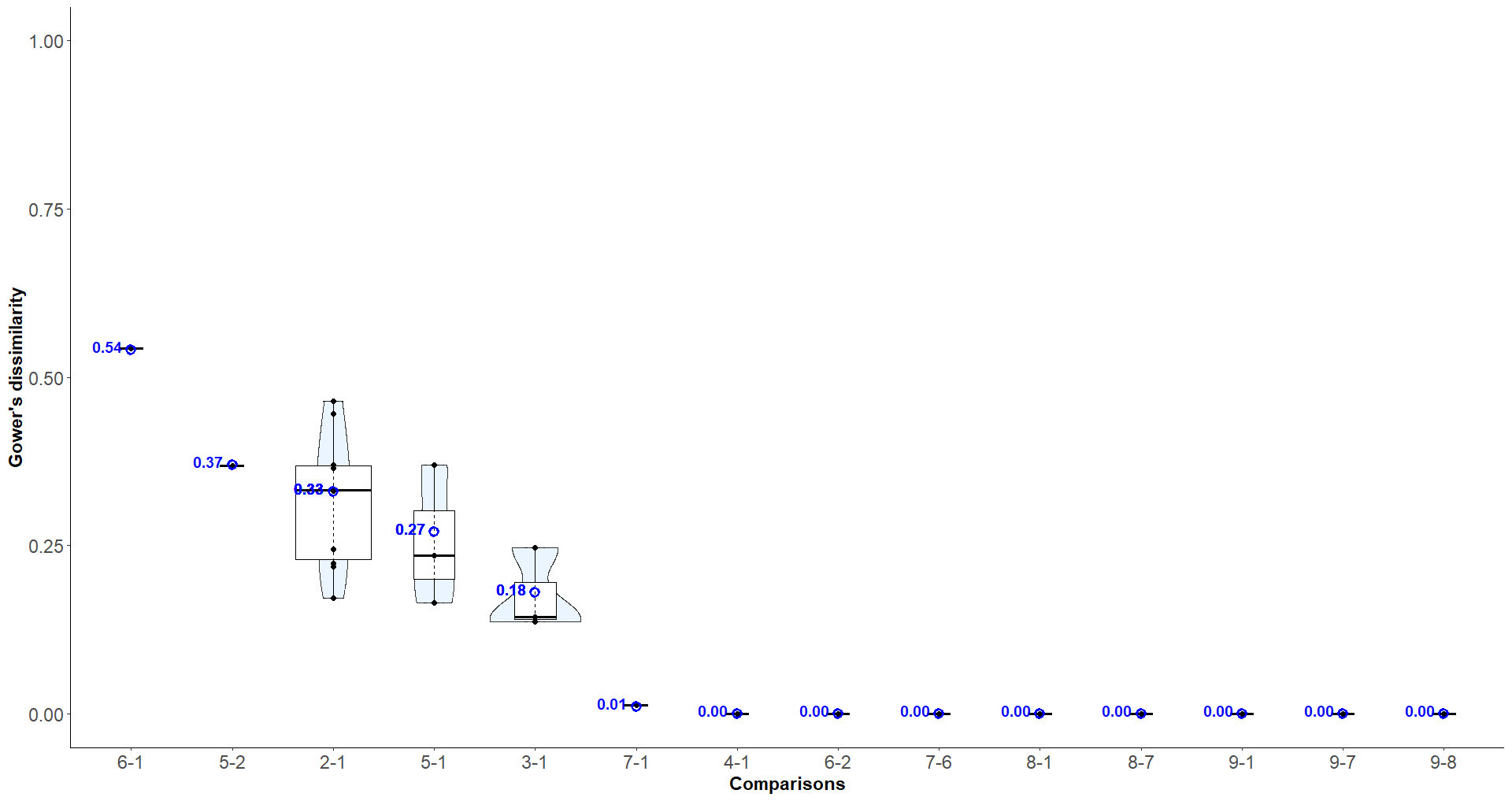


- violin plot with integrated box plots and dots for the *off-diagonal elements*, with each element (i.e., pair of comparisons) being a violin ($Between_comparison_dissimilarity). The plot is cluttered (and unpretty) in this example due to many pairs of comparisons:


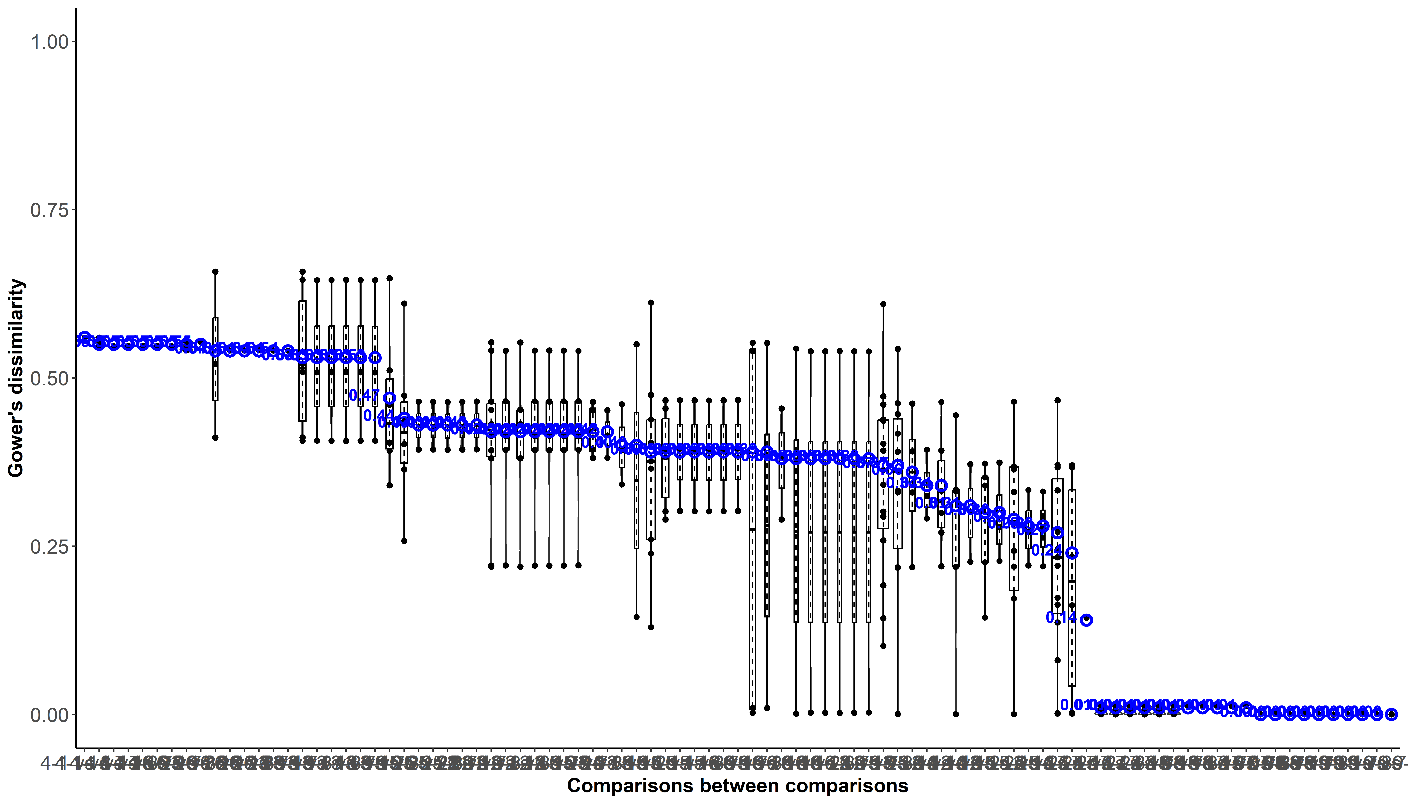


The second table is illustrated as a heatmap using the selected threshold of low dissimilarity ($Dissimilarity_heatmap). The observed comparisons appear on the rows and columns of the heatmap. We did not define the names of the treatments in the comp_clustering function; hence, the function used their ID number:


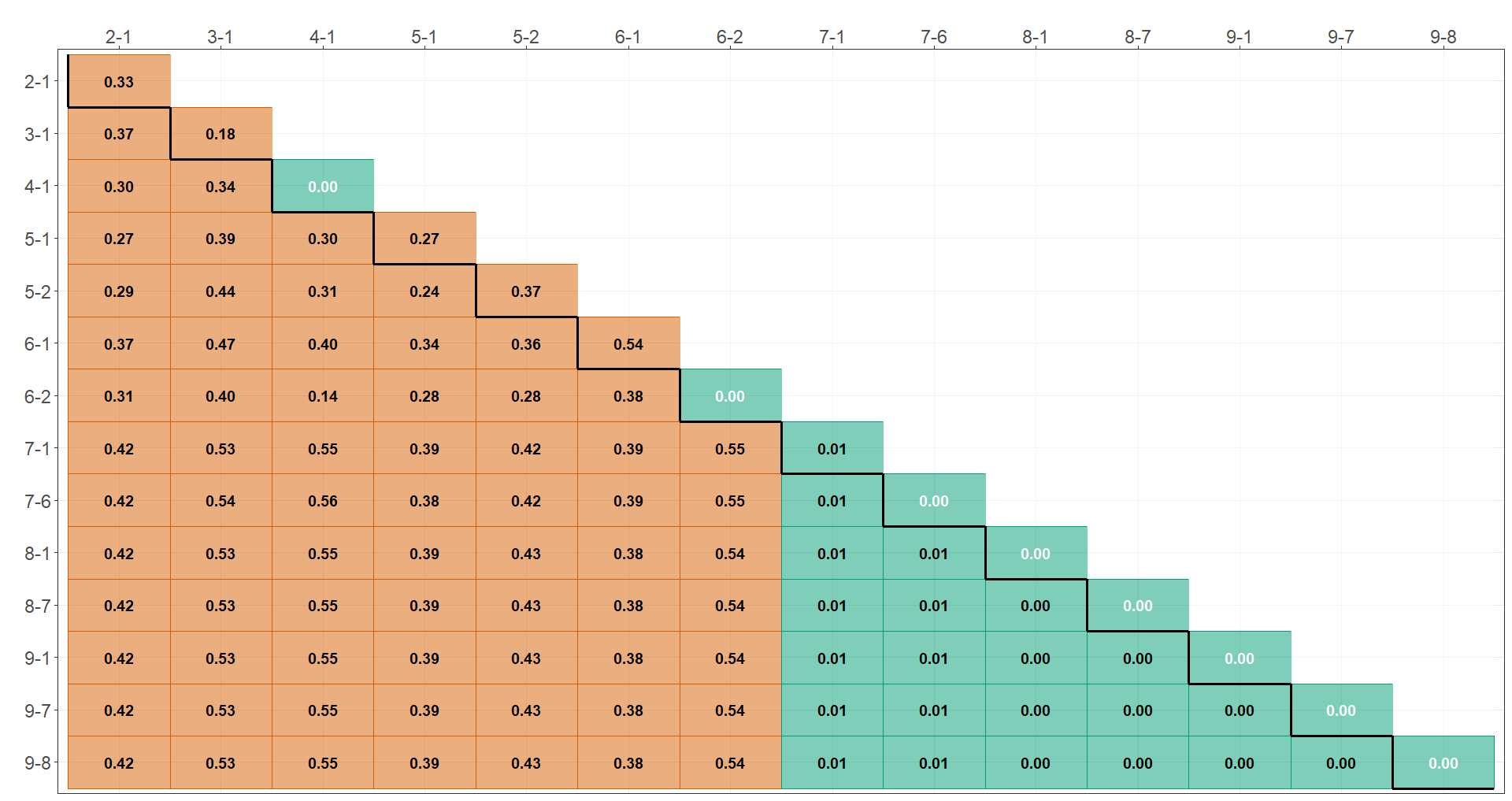


Further information in the console includes the type of the analysed characteristics ($Types_used) and the percentage of total missing data in the dataset ($Total_missing):

$Types_used

characteristic type

1 sex integer

2 sample.size double

3 h.rPTH integer

4 calcium double

5 vitamin.D double

6 duration double

7 quality double

$Total_missing

[1] "1.14%"

Refer to the official repository of rnmamod (<https://loukiaspin.github.io/rnmamod/>) to navigate to the functionalities and output of this R package.

**Interpretation**

The GD metric for the within- and between-comparison dissimilarity covered a great range of values, indicating variability in the distribution of the characteristics within and between comparisons. The heatmap presents the between-comparison dissimilarity for all possible pairs of observed comparisons in the network and aids in judging the transitivity. Redish cells below the main diagonal refer to pairs of comparisons with larger average GD values than the threshold of low dissimilarity (here, specified at 0.13), pointing to potential intransitivity. Greenish cells (below the main diagonal) indicate low to negligible average GD values for the corresponding pairs of comparisons. The heatmap helps identify ‘spots’ of potential intransitivity in the network. The study discusses the selection of thresholds of low dissimilarity [1].

**References**

1. Spineli LM. An empirical study on 209 networks of treatments revealed intransitivity to be common and multiple statistical tests suboptimal to assess transitivity. *BMC Med Res Methodol*. 2024;24(1):301.
2. Vestergaard P, Jorgensen NR, Mosekilde L, Schwarz P. Effects of parathyroid hormone alone or in combination with antiresorptive therapy on bone mineral density and fracture risk--a meta-analysis. *Osteoporos Int*. 2007;18(1):45-57.
3. Spineli LM, Kalyvas C, Yepes-Nuñez JJ, García-Sierra AM, Rivera-Pinzón DC, Seide SE, et al. Low awareness of the transitivity assumption in complex networks of interventions: a systematic survey from 721 network meta-analyses. *BMC Med*. 2024;22(1):112.
4. Schwarz EI, Scherff F, Haile SR, Steier J, Kohler M. Effect of Treatment of Central Sleep Apnea/Cheyne-Stokes Respiration on Left Ventricular Ejection Fraction in Heart Failure: A Network Meta-Analysis. J Clin Sleep Med. 2019;15(12):1817–25..
5. Sbidian E, Chaimani A, Guelimi R, Garcia-Doval I, Hua C, Hughes C, et al. Systemic pharmacological treatments for chronic plaque psoriasis: a network meta-analysis. *Cochrane Database Syst Rev*. 2023;7(7):CD011535.
6. Spineli LM. rnmamod: Bayesian Network Meta-analysis with Missing Participants. R package version 0.4.0. 2024. <https://CRAN.R-project.org/package=rnmamod>.
