# Supplementary material for: A database to initiate methodological advances in the evaluation of transitivity assumption in network meta-analysis: qualitative features and limitations of the tracenma R package
Source: BMC Med Res Methodol. 2025 Jul 31;25:183. doi: 10.1186/s12874-025-02634-x (PMC12315375; doi:10.1186/s12874-025-02634-x)
Supplement: Supplementary file 3 — Additional file 3. Distribution of the number of characteristics across datasets in the tracenma database. Figure S2. Box plots with integrated dots on the percentage of characteristics with missing data across all datasets, distinguishing among the clinical and methodological characteristic subtypes [file 12874_2025_2634_MOESM3_ESM.docx]

**Supporting Information for the article 'A database to initiate methodological advances in the evaluation of transitivity assumption in network meta-analysis: qualitative features and limitations of the tracenma R package'**

Loukia M. Spineli^1^  [Spineli.Loukia@mh-hannover.de](mailto:Spineli.Loukia@mh-hannover.de)

Andrés Mauricio García-Sierra^2^ [andresmauriciog@uchicago.edu](mailto:andresmauriciog@uchicago.edu)

Juan Jose Yepes-Nuñez^3,4^  [jj.yepesn@uniandes.edu.co](mailto:jj.yepesn@uniandes.edu.co)

^1^Midwifery Research and Education Unit, Hannover Medical School, Hannover, Germany

^2^Department of Public Health Sciences, University of Chicago, Chicago, USA

^3^School of Medicine, Universidad de los Andes, Bogotá D.C., Colombia

^4^Internal Medicine Department, Fundación Santa Fe de Bogotá, Bogotá D.C., Colombia

**
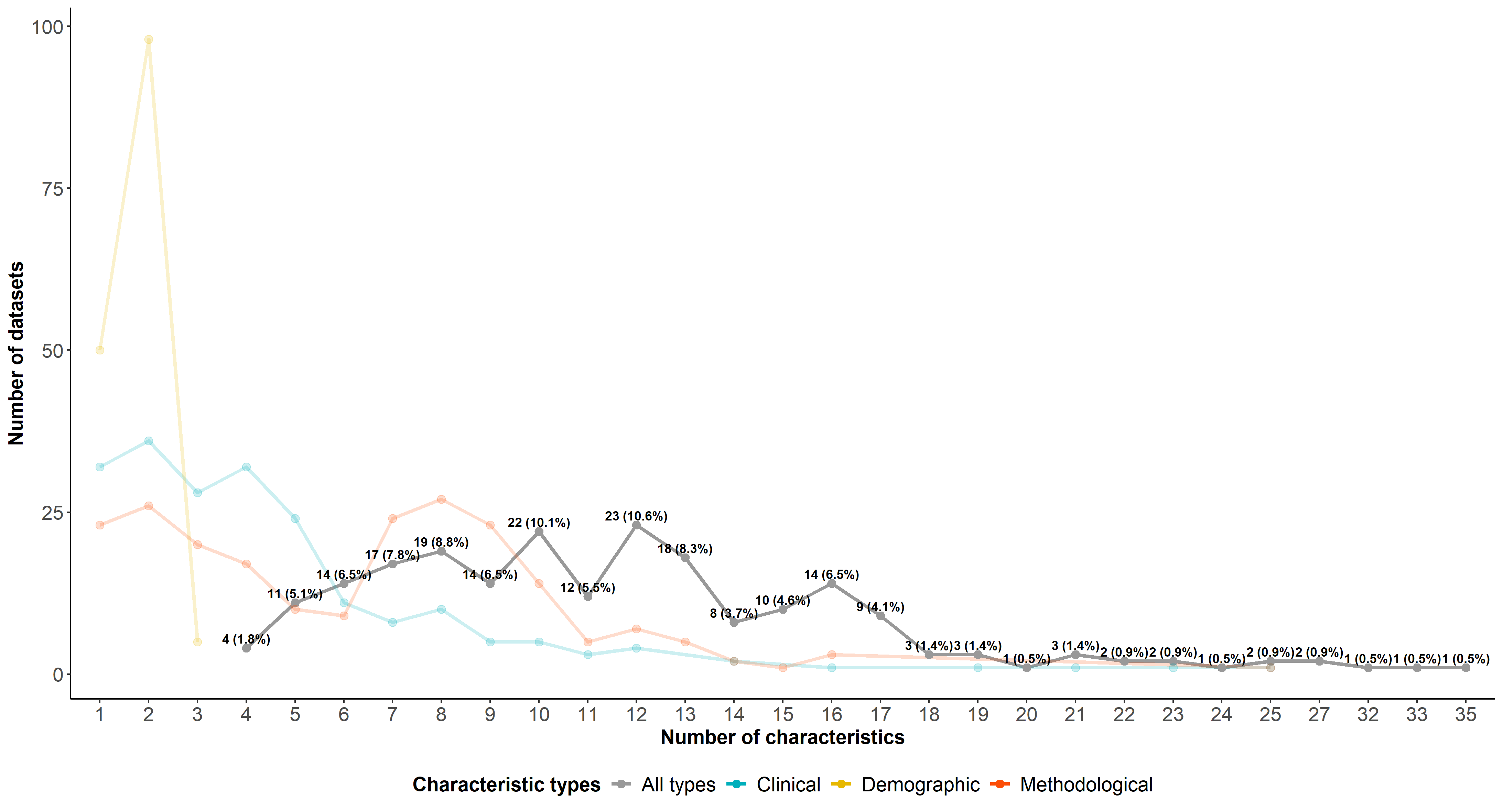
Figure S1.** Line plot on the number (and percentage) of datasets that include a specific number of characteristics (x-axis) regardless of the characteristic type (grey line), and based on the characteristic type: clinical, demographic and methodological. Emphasis is placed on all types of characteristics (grey line), using more opacity and not depicting the number and percentage of datasets for the lines referring to the characteristic types.

**
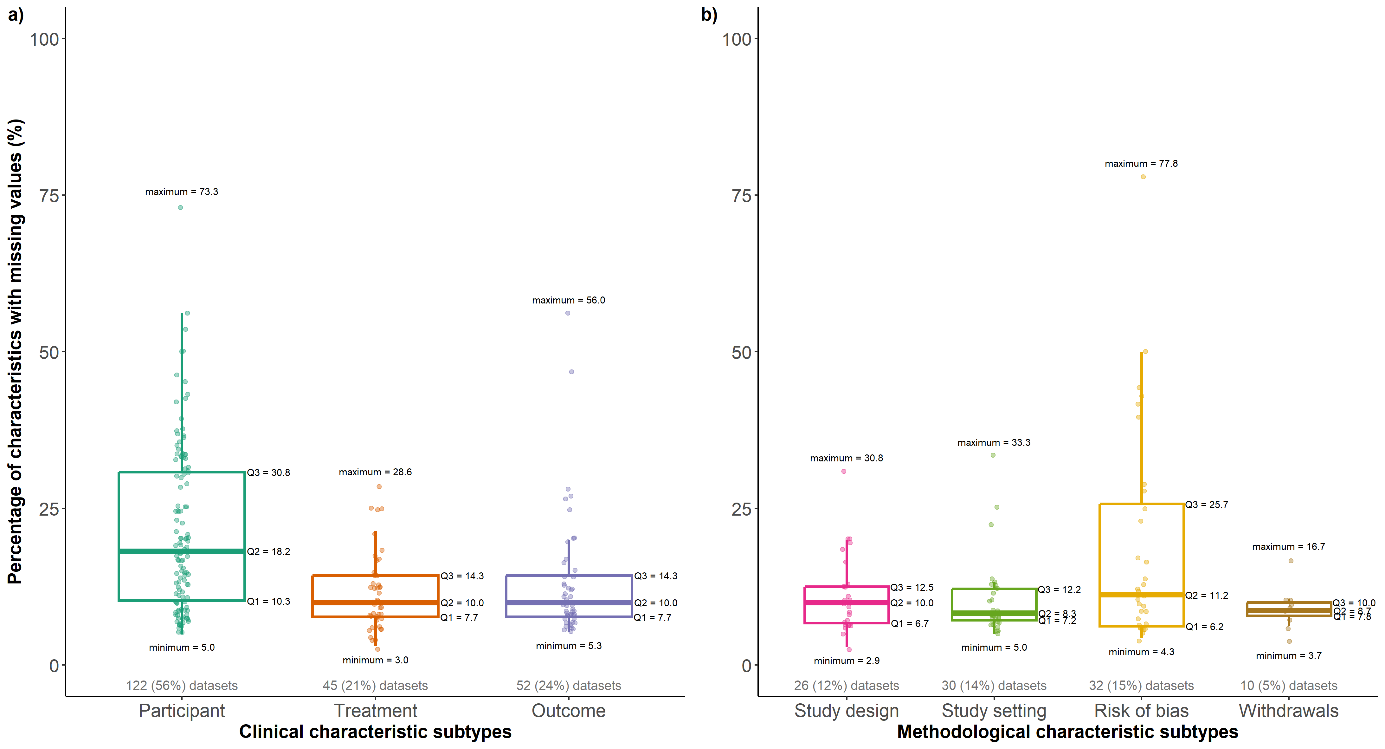
Figure S2.** Box plots with integrated dots on the percentage of characteristics with missing data across the 217 datasets, distinguishing among the three clinical characteristic subtypes (plot a)) and four methodological characteristic subtypes (plot b)). Five quartiles accompany all box plots, including the minimum, the first quartile (Q1), the median (Q2), the third quartile (Q3), and the maximum. The number and percentage of corresponding datasets (out of 217) appear in grey below the box plots.
